# Influence of Plant Developmental Phase and Irrigation Level on Cultivable Microbiome of Maize Root

**DOI:** 10.3390/biology14121694

**Published:** 2025-11-28

**Authors:** Carina Sá, Clarisse Brígido, Cátia Fidalgo, Adília Pires, Artur Alves, Etelvina Figueira, Paulo Cardoso

**Affiliations:** 1 CESAM, Centre for Environmental and Marine Studies and Department of Biology, University of Aveiro, 3810-193 Aveiro, Portugal; carinafsa@ua.pt (C.S.); cifidalgo@ua.pt (C.F.); adilia@ua.pt (A.P.); artur.alves@ua.pt (A.A.); efigueira@ua.pt (E.F.); 2 MED Mediterranean Institute for Agriculture, Environment and Development & CHANGE Global Change and Sustainability Institute, Universidade de Évora, Largo dos Colegiais 2, 7004-516 Évora, Portugal; clarisse.brigido@esa.ipsantarem.pt; 3 Santarém Polytechnic University, School of Agriculture, Quinta do Galinheiro, S. Pedro, 2001-904 Santarém, Portugal

**Keywords:** plant growth-promoting bacteria, drought, maize rhizosphere, maize endophytic bacteria, plant developmental phases

## Abstract

Plant growth-promoting bacteria can help plants survive in stressful environments. Here, we describe the isolation of root-surface and endophytic bacteria from maize roots at two different phases of the plant life cycle (vegetative and reproductive), grown under three different water regimes (100%, 50%, and 0%). Isolates were typed using BOX-PCR to identify unique genetic fingerprints, resulting in a total of 400 strains. These strains were screened for osmotic stress tolerance using 15% polyethylene glycol 6000. Isolates were also tested for bacterial plant growth-promoting traits, including the ability to produce siderophores, indole-3-acetic acid synthesis, and phosphate solubilization, both in the presence and absence of osmotic stress. The results showed that in the reproductive phase, a higher percentage of endophytic and rhizoplane bacteria were tolerant to osmotic stress. Additionally, the highest values of alginate and siderophore production by rhizoplane bacteria were also observed in the reproductive phase. These findings suggest that isolation of maize bacteria should consider the plant’s developmental phase and hydric stress conditions to effectively select bacterial strains that enhance crop resilience in drought-affected areas.

## Simple Summary

Water deficit affects crop productivity and the composition of root-associated microbial communities. Thus, it is important to understand the dynamics of bacterial communities during maize development and their response to water stress, shedding light on community adaptations. The analysis of the shifts on the ability of the bacterial community in improving plant development during water deficit is useful in the selection of biofertilizers. In this study, we observed shifts in the principal bacterial genera and in the community ability to improve plant growth during maize development in the presence of water stress. Our results give us insights into the best timing and conditions to select bacterial strains able to improve crop tolerance to stress.

## 1. Introduction

Global warming is expected to continue to increase and to affect climate systems, namely the water cycle, leading to an intensification of drought environments in several regions of the world [1]. The impact of more frequent and intense droughts has been felt in southern and western Europe, affecting water supply, agriculture, and sustainable energy production [2]. Water deficits negatively influence the morphological and biochemical processes in plants, bacterial communities, and their interactions [3,4,5,6,7].

Bacteria play important roles in the soil. They break down organic matter, contributing to nutrient recycling [8,9]. However, water availability affects soil microbial activity. When exposed to water deficit, bacteria have difficulty thriving and may form stable aggregates that improve the water permeability and water-holding capacity of the soil [10,11,12]. The creation of symbiotic interactions between bacteria and plants improves plant development [13,14]. Plant growth-promoting bacteria (PGPB) can facilitate nutrient absorption (N, Fe, P, and K), modulate phytohormone levels, induce systemic resistance, and produce and release a variety of metabolites and bioactive compounds such as lipopeptides, bacteriocins, antibiotics, biosurfactants, microbial volatile compounds, osmolytes, antioxidants, and phytohormones, demonstrating bacterial biocontrol and stress-relieving capabilities [15,16,17,18,19].

Maize is one of the most important sources of calories [20] and one of the most produced cereals worldwide, with an expected increase in production of 165 million tons by 2032 according to the OECD Agriculture Statistics World Cereal Projections [21]. The importance of maize in food security has motivated the development of strategies to improve performance under drought conditions; for example, using genetic engineering, testing and developing hybrid maize tolerant to drought, or applying PGPB to improve soil conditions, increase nutrient availability, or produce bioactive compounds that interfere with plant development [22,23]. PGPB can interact with maize plants through extracellular or intracellular interactions, thereby influencing maize development [24]. Some genera associated with maize roots have the ability to fix nitrogen; produce indole acetic acid (IAA) and siderophores; solubilize calcium phosphate, calcium phytate, and potassium; and prevent the proliferation of fungal pathogens such as *Fusarium graminearum*, *Rhizoctonia solani*, and *Exserohilum turcicum* [25,26]. Several studies have shown that the application of bacteria increases maize growth in greenhouse and field trials, with better results observed for bacterial combinations [27,28,29,30]. PGPB can also benefit maize plants under stressful conditions by increasing nutrient availability and improving water absorption [31,32,33].

Plant presence can influence the occurrence of PGPB in the soil [34]. This influence is primarily mediated through root exudates, which directly impact the composition of microbial communities in the rhizosphere [35,36]. Investigation into maize-associated microbial communities revealed variations in the microbial community associated with the rhizosphere throughout the maize life cycle, indicating adaptation of the microbiome to the needs of the crop [37]. A study by Cavaglieri et al. [38] observed the presence of strains belonging to the genera *Bacillus*, *Arthrobacter*, *Azotobacter*, *Listeria*, *Micrococcus*, *Pseudomonas*, *Agromyces*, and *Sporolactobacillus*, but only *Bacillus*, *Arthrobacter*, and *Azotobacter* appeared at all maize plant growth phases in the rhizoplane. Kong et al. [39] demonstrated variations in the root-associated bacterial community across different maize varieties, but certain genera (*Sphingomonas*, *Pseudomonas*, *Stenotrophomonas*, *Buttiauxella*, and *Curtobacterium*) were present in all three varieties studied. Soil type is also known to influence root endophytic bacterial diversity. A study reported the functional role of soil characteristics in culturable endophyte diversity, identifying *Bacillus*, *Streptomyces*, *Paenibacillus*, and *Pseudomonas* as the common genera isolated from maize roots grown in three different soil types [25].

Water deficit also influences the bacterial communities associated with plant roots. Under drought conditions, the rhizosphere often becomes enriched with drought-tolerant taxa such as Actinobacteria and Firmicutes, while moisture-sensitive groups like Proteobacteria and Bacteroidetes tend to decline [3,40]. Ibarra et al. [41] observed that a decrease in irrigation regimes (100% and 30%) affected the bacterial community diversity of the maize rhizosphere. Alterations in fungal diversity have also been observed in the maize rhizosphere exposed to water stress [42]. A study by Carter et al. [43] demonstrated that inoculating maize with a microbiome derived from water-stressed soils enhanced the plant’s adaptability under drought conditions. However, the interaction between irrigation and plant development in shaping bacterial functional traits and the mechanisms driving these changes remain poorly understood. The lack of such integrated studies limits our capacity to predict and manage the functional outcomes of microbes in agroecosystems under varying water regimes and crop developmental timelines. This gap is particularly pressing in the context of climate change and water-use efficiency, where synchronizing irrigation strategy with plant development and microbial functional windows could enhance crop performance and microbial services. Filling this gap will advance microbiome-informed agronomy in water-limited systems.

Given the integral role of bacterial–plant interactions, not only in plant development but also in the establishment of soil microbial communities, it is expected that water stress will affect both plant development and the composition of associated bacterial communities. Changes in the soil bacterial communities induced by stress can lead to changes in the mechanisms that these communities use to cope with stress, leading to the question of how the activation of specific survival mechanisms influences plant growth-promoting capacity? This study aimed to assess the changes that occur in the cultivable bacterial community associated with maize roots under water stress, to observe whether these bacterial communities become more resistant to osmotic stress throughout the plant’s developmental cycles, and to determine whether they can maintain their plant growth promotion characteristics when exposed to osmotic stress.

## 2. Materials and Methods

### 2.1. Growth of Maize with Different Irrigation Levels

*Zea mays* (DKC6092), growing in Estação Experimental António Teixeira, Coruche, Santarém, Portugal (38.942952, −8.512521), was used to isolate bacteria in the present study.

**Figure 1:**
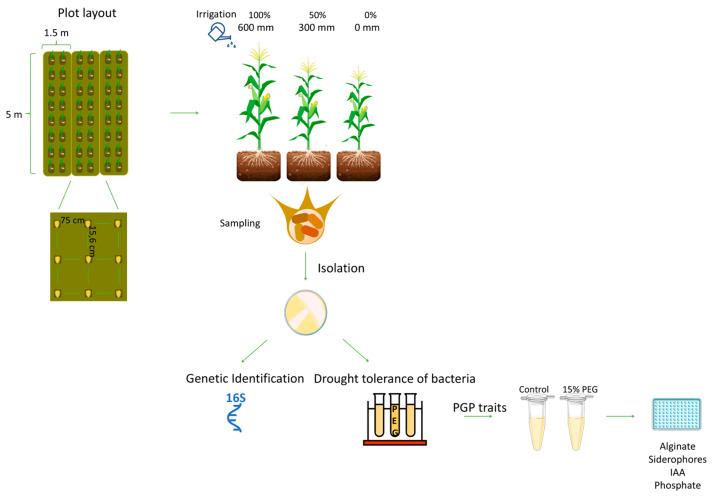
Schematic representation of the study. Plot layout, maize exposed to different irrigation regimes, isolation of bacteria associated with roots, bacterial genetic identification, bacterial osmotolerance evaluation, and analysis of the plant growth promotion abilities of isolates under control and osmotic stress (15% polyethylene glycol (PEG) 6000).

The soil at the study site is classified as sandy loam due to its composition (11% clay, 21.5% silt, and 67.5% sand, with a pH of 6.07). According to meteorological data registered by the Automatic Meteorological Station of Coruche (38°56′29.8″ N, 8°30′47.2″ W), located at the experimental site, a total precipitation of 76.2 mm, an average temperature of 18.9 °C per day, and an average evapotranspiration of 901.0 mm (5.30 mm/day) were observed during the experiment (14 May to 30 September 2021). For more details regarding soil physical–chemical properties and meteorological conditions, please consult Sá et al. [44]. Maize was cultivated in three plots, each 5 m long and 1.5 m wide. Maize was sown along the entire length of the plots using a tractor. The seeds were sown 15.6 cm away from each other with row spacing of 0.75 m (Figure 1). The seeds were sown on 14 May 2021, and germinated on 21 May 2021.

To isolate root-associated bacteria under different water availability conditions, maize plots were subjected to three irrigation regimes: one plot with 100% irrigation (600 mm; control), the second plot with 50% irrigation (300 mm), and the third plot with no irrigation. Line drip irrigation was initiated on 26 June 2021, owing to climatic conditions. The irrigation system consisted of a watering tube (22 mm in diameter) with drippers placed 20 cm apart and a water debit of 1.1 L/h. During the two selected sampling times, the development of the maize plants was evaluated: during the vegetative phase, when the plants had 12 leaves completely unfolded, stems in the process of elongation, and five internodes formed (13 July 2021), and during the reproductive phase, when the plants had a stem completely elongated, nine internodes formed, a flag leaf present, a tassel fully formed, and two ears with husk and silks dried out (24 August 2021). For each time point, five plants were randomly collected per plot.

### 2.2. Cultivable Bacterial Community Associated with Maize Roots

#### 2.2.1. Bacteria Living Inside and on the Surface of Roots

To study the bacterial communities associated with the maize root systems, bacteria were isolated from the interior (endophytic) and root surface (rhizoplane). The sampled roots were individually washed with tap water to remove the excess sediment, after which three root segments (1 cm) were cut from each root for bacterial isolation. The segments were then washed with sterile deionized water and analyzed individually. Each segment was rubbed onto a plate with yeast mannitol agar (YMA) medium (1 g mannitol (Sigma-Aldrich, Burlington, MA, USA), 0.5 g of K_2_HPO_4_ (Fisher BioReagents, Waltham, MA, USA), 0.2 g of MgSO_4_·7H_2_O (Sigma-Aldrich, USA), 0.1 g of NaCl (Sigma-Aldrich, USA), 0.5 g of yeast extract (Alfa Aesar, Heysham, UK), 1 L of dH_2_O, and 15 g of agar (Liofilchem, Roseto degli Abruzzi, Italy), adjusted to pH 6.8 [45] to obtain the bacteria associated with the roots. The same segments were sterilized externally through a new passage of deionized water, 96% ethanol for 5 s, and 3% hydrogen peroxide for 2 min, and finally washed with sterile deionized water to remove traces of the reagents [45]. At the end of the sterilization process, the root segments were crushed using a sterile toothpick to access the endophytic bacteria. Using the toothpick embedded in the internal contents of the root, four streaks were made on a plate containing YMA medium. Morphologically distinct colonies were subcultured on new plates. Plates were stored at 4 °C to inhibit proliferation. Isolated colonies were preserved in glycerol and frozen at −80 °C.

#### 2.2.2. Bacterial Numbers Present in the Rhizosphere of Maize Roots

To estimate the number of bacteria associated with the maize rhizosphere, soil attached to the roots of five maize plants from the vegetative and reproductive phases, exposed to three irrigation conditions, was collected. To determine the colony forming units (CFUs), the soil was homogenized, and 1 g of soil was placed in a centrifuge tube (15 mL) with 9 mL of sterile distilled water, and three technical replicates were performed of each sampled soil. The samples were subjected to orbital agitation at 150 rpm for 1 h using a GFL Orbital Shaker 3005 (GFL Gesellschaft für Labortec, Munich, Germany). The soil was allowed to settle, and 1 mL was diluted in 9 mL of sterile distilled water and stirred to homogenize the solution. Successive dilutions were prepared until a 10^−5^ dilution was obtained. The obtained dilutions were inoculated into YMA medium by surface incorporation using 100 µL of the dilutions. The solution was evenly distributed over the plate using a spreader, and three independent replicates were performed. After inoculation, the plates were inverted and placed in an incubator at 26 °C for 2–9 days, as described by Vincent et al. [46].

### 2.3. Bacteria Identification

#### 2.3.1. PCR-Based Fingerprinting

In total, 617 bacterial isolates obtained from the inside and rhizoplane of the roots were typed using BOX-PCR fingerprinting.

Isolates were inoculated on YMA, and single colonies were used to prepare a bacterial suspension in 20 µL molecular-grade TE buffer. The tubes were heated on a hotplate for 5 min at 100 °C to lyse cells. Each PCR reaction (total volume: 25 μL) tube contained a mixture of 1 μL bacterial suspension, 2 μL BOXA1R primer (5′-CTACGGCAAGGCGACGCTGAC-3′) [47], which was diluted in PCR-grade water to 10 μmol/μL, 12.5 μL NZYTech 2X Taq Green Master Mix (NZYTech, Portugal), and 15.75 μL PCR-grade water.

Amplification was performed using one cycle at 95 °C (7 min), followed by 30 cycles at 94 °C (1 min), 53 °C (1 min), and 65 °C (8 min), and a final step at 65 °C (16 min). Ten microliters of each PCR product and 3 µL of a molecular weight ladder (NZYDNA Ladder III, 200–10,000 bp, NZYtech, Lisbon, Portugal) were loaded onto a 1% agarose gel and electrophoresed in TAE buffer at 80 V for 60 min. Agarose gels were stained with ethidium bromide and scanned with Bio-Rad GelDoc System (Bio-Rad, Hercules, CA, USA).

Using Pearson’s correlation coefficient and unweighted pair group method with arithmetic mean (UPGMA) grouping of averaged profile similarities, GelCompar II (Applied Maths, Sint-Martens-Latem, Belgium) was used to assess the PCR fingerprinting profiles. Clusters were created using profiles with similarity values higher than 92.3%. A representative isolate from each cluster was randomly selected for 16S RNA gene sequencing (total of 400 representative isolates).

#### 2.3.2. 16S rRNA Gene Amplification and Phylogenetic Analysis

Extraction of total DNA for each isolate was performed as described by Rivas et al. [48].

The 16S rRNA gene of the representative isolates selected based on PCR-based fingerprinting was amplified using primers 799F (5′-AAC MGG ATT AGA TAC CCK G-3′) [49] and 1492R (5′-GGT TAC CTT GTT ACG ACT T-3′) [50]. Each PCR reaction (20 μL final volume) contained 1 μL of template DNA (~10 ng), 10 μL of DreamTaq Green PCR Master Mix (2×) (Thermo Scientific, Waltham, MA, USA), and 7.5 pmoles of each primer (799F/1492R). The amplification program consisted of an initial denaturation at 95 °C for 3 min, followed by 35 cycles of denaturation at 94 °C for 30 s, annealing at 58 °C for 40 s, and extension at 72 °C for 30 s, and a final extension step at 72 °C for 10 min.

For some isolates, amplification was performed using the primer pair 799F and 1391R (5′-GAC GGG CGG TGW GTR CA-3′) [51], using the same reaction mix described above. The PCR cycling conditions were as follows: initial denaturation at 95 °C (3 min), 35 cycles of denaturation at 94 °C (45 s), annealing at 56 °C (45 s), and extension at 72 °C (45 s), followed by a final extension at 72 °C (10 min).

PCR products were purified using the DNA Clean & Concentrator or Zymoclean^TM^ Gel DNA Recovery kits (Zymo Research, Freiburg, Germany) and sent to Eurofins Genomics (Ebersberg, Germany) for Sanger sequencing using the 799F primer. Sequences were edited using FinchTV V1.4.0 software. A BLAST (2.16.0) search against the GenBank and EzBioCloud databases was performed to identify bacteria at the family and genus levels [52,53]. Partial 16S rRNA gene sequences for the majority of representative isolates were deposited in GenBank (OR948054-OR948403; PP150899-PP150922).

### 2.4. Osmotolerance and Plant Growth Promotion Abilities of Strains Exposed to Osmotic Stress

#### 2.4.1. Osmotolerance of Bacterial Strains

Representative isolates (400) were grown in microtubes (1.5 mL) containing 500 µL of yeast broth mannitol (YMB) medium [45] supplemented or not (control) with polyethylene glycol (PEG) 6000 (TCI, Tokyo, Japan) at 15% (osmotic potential of the media was −0.040 and −0.70 MPa [54], respectively). The tubes were inoculated with 50 µL of bacterial culture and incubated at 27 +/− 1 °C in an Orbital Shaker-Incubator ES-20 (Grant-bio, Nottingham, UK), (150 rpm) for 48 h. Growth was determined by measuring optical density at 600 nm using a Tecan Infinite 200 Pro microplate reader (Tecan Group Ltd., Männedorf, Switzerland). The relationship between optical density and cell concentration was obtained using the formula OD_600_ = 1.0 = 54.13 × 10^7^ cells/mL for OD_600_ < 0.3, and the formula y × 10^7^ cells/mL = (OD_600_ × 85.53) − 9.42 for OD_600_ ≥ 0.3. Cell concentration was expressed as the number of cells per milliliter (number of cells/mL). Two independent experiments were performed with three replicates per condition. Osmotolerance was determined by Equation (1):

(1) $$Osmotolerance\;\left(\%\right)=\frac{15\%\;PEG\;growth-0\%\;PEG\;growth}{0\%\;PEG\;growth}\times 100\%$$

The medium from each tube was collected separately after centrifugation at 10,000× *g* for 10 min at 25 °C, using a Heraeus Multifuge 3S-R refrigerated centrifuge (Heraeus, Hanau, Germany), for extracellular alginate quantification.

#### 2.4.2. Determination of Extracellular Alginate

Alginate determination was adapted from the method described by Johnson et al. [55], using dimethyl methylene blue (DMB). DMB (100 μL) was added to 100 μL of the centrifuged growth medium (Section 2.4.1). Absorbance was immediately measured at 525 nm, and standards (1.25 to 25 μg/mL) were made with sodium alginate (Sigma-Aldrich, USA). The results are expressed in nanograms of alginate per cell (ng/cell).

#### 2.4.3. Determination of Indole-3-Acetic Acid

Indole-3-acetic acid (IAA) determination was adapted from the method described in Gordon and Robert [56] by supplementing cultures with tryptophan (5 mg/mL) (Prolabo-VWR, Radnor, Pennsylvania, USA) [57]. After incubation (48 h) the tubes were centrifuged at 10,000× *g* for 10 min at 25 °C, and the supernatant was collected. To 100 μL of the supernatant, 200 μL of Salkowsky reagent (0.5 M FeCl_3_ (Merck, Darmstadt, Germany) and 35% perchloric acid (Merck, Germany) were added. After incubation for 25 min at room temperature (23 °C), absorbance was measured at 530 nm using IAA standards (5–100 μg/mL) (Sigma-Aldrich, USA). The results are expressed as ng IAA per cell (ng/cell).

#### 2.4.4. Production of Siderophores

Siderophore production was analyzed as previously described in Arora [58]. After incubation (48 h) the tubes were centrifuged, at 10,000× *g* for 10 min at 25 °C, and the supernatant was collected. To 130 μL of the supernatant, 20 μL of chrome azurol S (CAS) solution (50 mL of CAS (Riedel-deHaen, Charlotte, NC, USA, EUA) (1.21 mg/mL), 10 mL of 1 mM FeCl_3_.6H_2_O (Merck, Germany) (in 10 mM HCl (Fisher Scientific, USA), and 40 mL of hexadecyltrimethylammonium bromide (HDTMA) (Sigma-Aldrich, USA) solution (1.82 mg/mL) were added to determine siderophore production. After incubation for 20 min at room temperature (23 °C), absorbance was measured at 630 nm. The percent siderophore units (*psu*) were calculated using the following Equation (2):

(2) $$psu=\frac{Ar-As}{Ar}$$

where Ar refers to the absorbance of the reference (CAS solution in uninoculated YMB), and As is the absorbance of the sample (CAS solution in inoculated YMB). The results are expressed as psu.

#### 2.4.5. Solubilization of Phosphate

Bacteria were grown in microtubes (1.5 mL) containing Pikovskaya liquid medium (0.5 mL) and were incubated at 26 °C for 10 days. The Pikovskaya medium consisted of 10 g of glucose (Sigma-Aldrich, USA), 5 g of Ca_3_(PO_4_)_2_ (Fluka, Buchs, Switzerland), 0.5 g of (NH_4_)_2_SO_4_ (Sigma-Aldrich, USA), 0.2 g of NaCl (Sigma-Aldrich, USA), 0.1 g of MgSO_4_·7H_2_O (Sigma-Aldrich, USA), 0.2 g of KCl (Merck, Germany), 0.5 g of yeast extract, 0.002 g of MnSO_4_·H_2_O (Sigma-Aldrich, USA), and 0.002 g of FeSO_4_·7H_2_O (Merck, Germany) in 1000 mL, at pH 7 [59,60]. After 10 days, the tubes were centrifuged at 13,000× *g* for 10 min at room temperature and the supernatant was preserved for phosphate quantification.

The determination of phosphate was adapted from a previously described method in Murphy [61]. In a microplate, 240 μL of supernatant, 12 μL of H_2_O, 48 μL of reagent solution sulfuric acid (Labkem, Dublin, Ireland) (1.25 M), and 37.5 mL of ammonium molybdate (Acros Organics, Geel, Belgium) (0.005 M), ascorbic acid (Sigma-Aldrich, USA) (0.03 M), and potassium antimonyl tartrate (Acros Organics, Belgium) (0.05 mg/mL) were added. Absorbance was measured at 882 nm after incubation for 10 min at room temperature (23 °C), with potassium dihydrogen phosphate (Merck, Germany) as a standard (0.04 to 3.45 mg/mL). The results are expressed in milligrams of phosphate per milliliter (mg P/mL).

### 2.5. Statistical Analysis

Statistical analysis (Tables S1–S3) was performed using permutational multivariate analysis of variance (PERMANOVA) with 9999 Monte Carlo tests using PRIMER 6 and PERMANOVA+ [62,63]. Significant differences were considered for *p* ≤ 0.05 and are identified in the figures with different lowercase letters (vegetative phase or control condition), uppercase letters (reproductive phase or osmotic stress), and asterisks (between conditions for the same percentage of irrigation).

## 3. Results

### 3.1. Colony Forming Units

**Figure 2:**
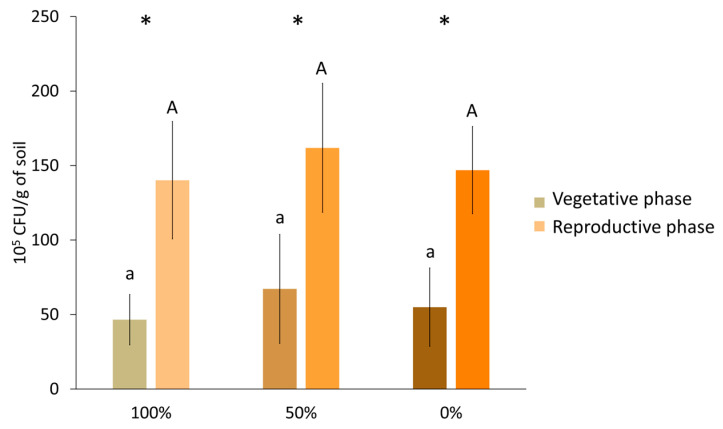
Rhizospheric colony-forming units (CFUs). The values are expressed as 10^5^ CFU/g of soil. Significant differences among irrigation conditions (100%, 50%, and 0%) are indicated by different lowercase and uppercase letters for the vegetative and reproductive phases, respectively (*p* < 0.05). Differences in the same level of irrigation between phases are indicated with an asterisk (*p* < 0.05).

In the vegetative phase, there was an increase (although not statistically significant) in colony forming units (CFUs) in the rhizosphere exposed to water stress: 44% in 50% irrigation and 18% in 0% irrigation compared to 100% irrigation (Figure 2, Table S4). A similar effect was observed in the reproductive phase; the number of cells found in the soil surrounding the roots increased (although not significantly) 15.5% in 50% irrigation and 5% in 0% irrigation. A significant increase in CFUs in the reproductive phase compared to the vegetative was observed across all irrigation conditions (201% under 100% irrigation, 141% under 50% irrigation, and 167.5% under 0% irrigation).

### 3.2. Bacterial Identification

In this study, 400 unique strains were detected by fingerprinting, and we were able to identify 374 by partial 16S rRNA gene sequences deposited in GenBank (Table S5). Certain strains are prevalent in more than one of the bacterial communities studied. In the vegetative phase, 31, 25, and 17 endophytic strains and 43, 44, and 30 rhizoplane strains were found in maize roots exposed to 100%, 50%, and 0% irrigation levels, respectively. In the reproductive phase, 40, 35, and 22 endophytic strains and 54, 52, and 47 rhizoplane strains were found in 100%, 50%, and 0% irrigation levels, respectively.

#### 3.2.1. Bacterial Strains from the Vegetative Stage

**Figure 3:**
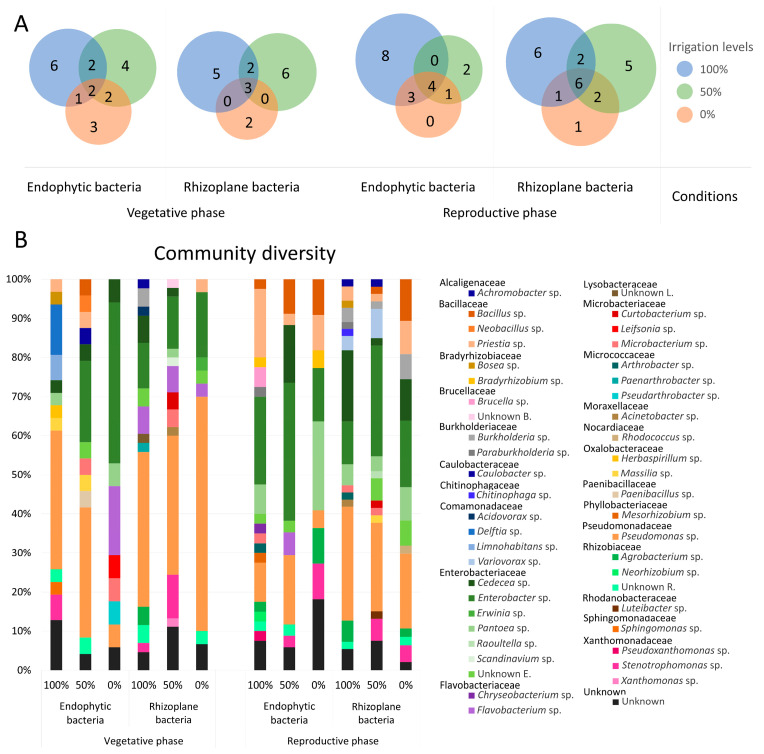
Diversity of cultivable bacterial communities. (**A**) Venn diagrams of the number of bacterial genera isolated outside (rhizoplane) and inside (endophytic) maize roots at two developmental phases (vegetative growth and reproductive growth), exposed to different levels of irrigation (100% blue, 50% green, 0% orange). (**B**) Relative abundance of the strains from each bacterial genus under each condition.

Based on the taxonomic identification of the bacterial isolates, during the vegetative phase, the endophytic bacterial community exhibited the presence of two genera, *Pseudomonas* and *Cedecea*, which were common to all levels of irrigation. Additionally, *Enterobacter* and *Microbacterium* were only detected under water-deficit conditions (50% and 0% irrigation). In the rhizoplane, three genera, *Pseudomonas*, *Flavobacterium*, and *Enterobacter*, were ubiquitously found across all irrigation treatments and no bacterial genera were shared exclusively between the water-deficit conditions (Figure 3A).

*Pseudomonas* was the most abundant genus of endophytic strains in the vegetative phase under both 100% and 50% irrigation conditions, accounting for 35.5% and 33% of the total isolates, respectively. Under extreme water-deficit conditions (0% irrigation), the relative abundance of *Pseudomonas* markedly decreased to 6%, while *Enterobacter* was the most abundant genus, representing 41% of the community (Figure 3B). The genera *Bosea*, *Delftia*, *Limnohabitans*, *Herbaspirillum*, *Sphingomonas*, and *Stenotrophomonas* were exclusively found in plots irrigated at 100%.

Water stress shifted the cultivated endophytic microbiome, with a reduction in *Pseudomonas* spp. and increases in Enterobacter spp., *Cedecea* spp., and *Microbacterium* spp., along with the increase in water stress level. Several genera were specific to certain irrigation treatments: *Bacillus*, *Neobacillus*, *Caulobacter*, and *Paenibacillus* were found only under 50% irrigation, while *Lavobacterium*, *Leifsonia*, and *Pseudarthrobacter* were exclusive to plots without irrigation (0%).

In the rhizoplane-associated cultivable bacterial community, *Pseudomonas* remained the predominant genus under all irrigation regimes, representing 39.5%, 35.5%, and 60% of the community under 100%, 50%, and 0% irrigation, respectively, followed by *Enterobacter* (11.63%, 13.33%, and 16.67%) and *Flavobacterium* (6.98%, 6.67% and 3.33%) (Figure 3B). The genera *Achromobacter*, *Burkholderia*, *Acidovorax*, *Paenarthrobacter*, and *Agrobacterium* and members of the *Lysobacteriaceae* family were detected only under 100% irrigation.

With increasing water stress, a decline in *Cedecea* spp. (6.98%, 2.22%, and 0%), and *Flavobacterium* spp. (6.98%, 6.67%, and 3.33%) was observed, while *Enterobacter* spp. increased progressively (11.63%, 13.33%, and 16.67%) with irrigation reduction. Additional genera were unique to specific treatments: *Pantoea*, *Scandinavium*, *Curtobacterium*, *Microbacterium*, *Acinetobacter*, and *Xanthomonas* were exclusive to 50% irrigation, whereas *Priestia* and *Erwinia* were only found under 0% irrigation.

#### 3.2.2. Bacterial Strains from the Reproductive Stage

In the reproductive phase, four endophytic genera, *Pseudomonas*, *Enterobacter*, *Priestia*, and *Bacillus*, were consistently detected across all irrigation levels. Only one genus, *Stenotrophomonas*, was shared exclusively between 50% and 0% irrigation. In the rhizoplane, six bacterial genera (*Pseudomonas*, *Pantoea*, *Enterobacter*, *Cedecea*, *Burkholderia*, and *Priestia*) were found across all levels of irrigation. Two genera (*Bacillus* and *Stenotrophomonas*) were detected in both 50% and 0% irrigation plots (Figure 3A).

A higher relative abundance of *Enterobacter* was observed in the endosphere of roots exposed to 100% and 50% irrigation (22.5% and 35%, respectively). Conversely, under 0% irrigation, *Pantoea* was the most represented genus (23%) (Figure 3B). Several genera, including *Brucella*, *Paraburkholderia*, *Chryseobacterium*, *Microbacterium*, *Arthrobacter*, *Mesorhizobium*, *Neorhizobium*, and *Pseudoxanthomonas*, were only found under 100% of irrigation. A reduction in the abundance of *Priestia* (17.5%, 2.94%, and 9.09 under 100%, 50%, and 0% irrigation, respectively) was observed under increasing osmotic stress, accompanied by a corresponding increase in *Bacillus* (2.5%, 8.82%, and 9.09%, respectively). Under stressful conditions, *Stenotrophomonas* appeared to be an endophytic genus associated with maize roots.

In the rhizoplane, a higher relative abundance of *Pseudomonas* sp. was observed under 100% and 0% irrigation (29% and 19%, respectively), while *Enterobacter* predominated under 50% irrigation conditions (28%) (Figure 3B). The genera *Bosea*, *Paraburkholderia*, *Chitinophaga*, *Arthrobacter*, and *Acinetobacter* were present only with full irrigation (100%). A decrease in the relative abundance of *Cedecea* (18.18%, 1.89% and 10.64%) and *Pseudomonas* sp. (29.09%, 22.64%, and 19.15%) was observed with increasing water stress. Several genera were exclusive to certain irrigation levels: *Raoultella*, *Curtobacterium*, *Massilia*, and *Luteibacter* were found only under 50% irrigation, while *Rhodococcus* was detected only under 0% irrigation.

### 3.3. Osmotolerance of Bacteria

#### 3.3.1. Endophytic Bacteria

The majority of the endophytic strains isolated under the different irrigation regimes (100%, 50%, and 0%) during both the vegetative and reproductive phases exhibited low tolerance to osmotic stress.

**Figure 4:**
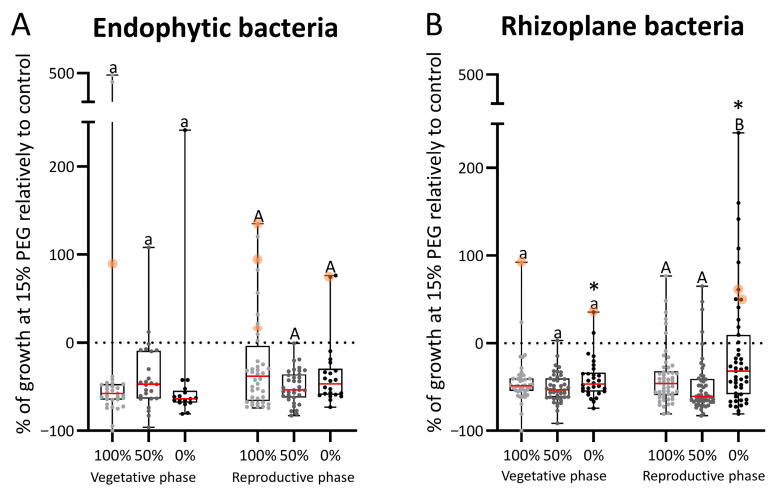
Osmotolerance of the bacterial strains. Bacterial growth at 15% polyethylene glycol (PEG) 6000 relative to the control (0% PEG). Bacteria were isolated from three types of irrigation (100% light gray, 50% medium gray, and 0% dark gray) during two different phases of maize development (vegetative and reproductive phases). The median of the distribution is indicated by the red line. Strains capable of using PEG 6000 as the carbon source are highlighted in light orange. (**A**) Endophytic bacteria. (**B**) Rhizoplane bacteria. Values represent the means of three to six replicates ± standard error. Significant differences among irrigation conditions (100%, 50%, and 0%) are indicated by different lowercase and uppercase letters for the vegetative and reproductive phases, respectively (*p* < 0.05). Differences in the same level of irrigation between phases are indicated with an asterisk (*p* < 0.05).

In the vegetative phase, only three endophytic strains isolated from plants under 100% irrigation exhibited increased growth under osmotic stress conditions induced by 15% PEG. Notably, PEG could be used as a carbon source by the strain with the smallest growth improvement (Figure 4A). Among the strains isolated under 50% irrigation, five demonstrated tolerance to osmotic stress, and two exhibited enhanced growth under stress conditions. From the 0% irrigation treatment, only one strain showed increased growth in response to osmotic stress. The strains with the highest tolerance were isolated from the 100% irrigation condition.

In the reproductive phase, 10 endophytic bacteria isolated from the 100% irrigation treatment exhibited enhanced growth under the presence of stress (15% PEG). Among these, three strains were able to use PEG as a carbon source (Figure 4A). Only one strain isolated from the 50% irrigation treatment in the reproductive phase could tolerate osmotic stress. Two strains isolated from 0% irrigation treatment in the reproductive phase showed increased growth under PEG-induced stress, one of which could use PEG as an energy source. The most tolerant strains were isolated from the 100% and 0% irrigation conditions.

#### 3.3.2. Rhizoplane Bacteria of Maize Roots

Most of the rhizoplane strains isolated from plants under different irrigation levels (100%, 50%, and 0%) in the vegetative and reproductive phases exhibited limited tolerance to osmotic stress. In the vegetative phase, only two rhizoplane bacteria isolated from 100% irrigation increased growth in response to osmotic stress (15% PEG), and one of these was able to use PEG as a carbon source (Figure 4B). Among strains isolated from plants under 50% irrigation, only one could tolerate osmotic stress, and at 0% irrigation, two strains enhanced growth under osmotic stress; of these, the strain with the highest relative growth was also able to metabolize PEG as a carbon source. No significant differences between irrigation levels were observed in the median relative growth under osmotic stress.

In the reproductive phase, six rhizoplane bacteria isolated under 100% irrigation increased growth in the presence of PEG (Figure 4B). Among the strains isolated under 50% irrigation, four could tolerate osmotic stress. Twelve strains isolated from plants under 0% irrigation increased growth in the presence of stress, two of which could use PEG as a source of energy. When comparing strains across irrigation treatments, those isolated under 0% irrigation exhibited significantly higher tolerance to osmotic stress than those in the other two conditions (100% and 50% irrigation). The most tolerant strains were consistently associated with the 0% irrigation condition.

A significant increase in drought tolerance was observed in the rhizoplane microbial community of plants subjected to 0% irrigation in the reproductive phase compared to the vegetative phase. Moreover, a higher number of stress-tolerant strains was observed in the reproductive phase across all irrigation treatments.

### 3.4. Production of Alginate

#### 3.4.1. Production of Alginate by the Endophytic Bacteria

**Figure 5:**
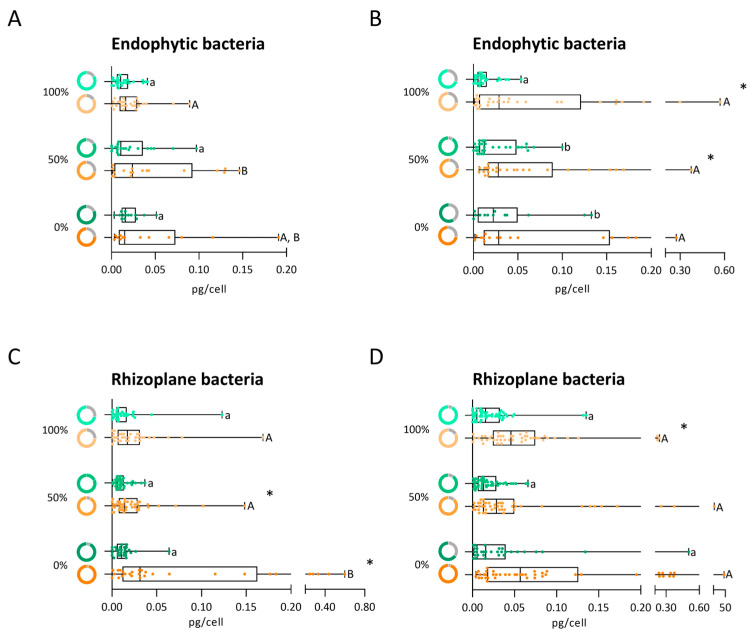
Alginate production by the bacterial strains in nanograms of alginate per cell (ng/cell). Control (green gradient) and osmotic stress (15% polyethylene glycol (PEG) 6000) (orange gradient) by bacteria isolated from the inside (endophytic) and outside (rhizoplane) of maize roots in two growth phases (vegetative and reproductive) exposed to three levels of irrigation (100%, 50%, and 0%). The median of the distribution is indicated by a black line, where the box represents positive values of the data. (**A**) Endophytic bacteria from plants in the vegetative phase, (**B**) endophytic bacteria from plants in the reproductive phase, (**C**) rhizoplane bacteria from plants in the vegetative phase, and (**D**) rhizoplane bacteria from plants in the reproductive phase. The respective donut charts represent the percentage of bacteria that are not capable (gray) or capable (green or orange) of alginate production. The boxplots include bacteria capable of producing alginate. Values are the means of three to six replicates ± standard error. Significant differences among levels of irrigation for control (0% PEG 6000) conditions are indicated with different lowercase letters, while for osmotic stress conditions (15% PEG 6000), they are indicated with different uppercase letters. Significant differences between osmotically stressed (15% PEG 6000) and non-stressed (0% PEG 6000) bacterial communities for each level of irrigation (100%, 50%, and 0%) are indicated by asterisks (*p* < 0.05).

In the vegetative phase under control conditions, the irrigation levels did not influence the percentage of endophytic bacteria capable of producing alginate, with similar frequencies observed across treatments (between 82% and 84%) (Figure 5A, green donut charts). The highest alginate producer (0.097 pg/cell) was isolated from roots exposed to 50% irrigation (Figure 5A, green boxplot). When the same bacterial community was exposed to osmotic stress, the percentage of alginate-producing strains declined with decreasing irrigation levels, from 84% under 100% irrigation to 72% and 76% under 50% and 0% irrigation, respectively (Figure 5A, orange donut charts). Despite the lower number of strains able to produce alginate under osmotic stress, the amount of alginate produced increased at all irrigation levels compared to control conditions (Figure 5A, orange boxplot).

In the reproductive phase, the irrigation level influenced alginate production of endophytic strains (70%, 91%, and 59% for 100%, 50%, and 0% irrigation, respectively) (Figure 5B, green donut charts). The median of alginate production increased with decreasing irrigation, and a significant difference was observed between 100% irrigation treatment and the two water stress conditions (Figure 5B, green boxplot). The highest alginate producer (0.13 pg/cell) was isolated from the 0% irrigation treatment. When the same bacteria were subjected to 15% PEG, the proportion of alginate-producing strains remained relatively stable across irrigation treatments (Figure 5B, orange donut charts). The highest alginate producers under osmotic stress were isolated from the 100% and 50% irrigation treatments (0.57 and 0.37 pg/cell, respectively) (Figure 5B, orange boxplot). A significant increase in alginate production was observed under stress compared to non-stress conditions for strains isolated from the 100% and 50% irrigation treatments.

Overall, alginate production by the endophytic bacterial community increased under osmotic stress in the reproductive phase compared to the vegetative phase, suggesting a potential role of alginate in stress adaptation.

#### 3.4.2. Production of Alginate by the Rhizoplane Bacteria

The decrease in irrigation increased the ability of bacteria to produce alginate (72%, 82%, and 93%) in the rhizoplane (Figure 5C, green donut charts). Although the median of alginate production was higher under 0% irrigation than the other conditions, no statistically significant differences were observed among the irrigation levels. The highest alginate producer (0.12 pg/cell) was isolated under 100% irrigation (Figure 5C, green boxplot). When the same bacterial community was exposed to osmotic stress, the proportion of alginate-producing isolates increased across all irrigation conditions (74%, 93%, and 97% under 100%, 50%, and 0%, respectively) (Figure 5C, orange donut charts). The bacterial community isolated from 0% irrigation showed a statistically significant increase relative to the control (0% of irrigation + 0% of PEG). Furthermore, in the 0% irrigation treatment, five bacterial strains exhibited the highest alginate production (ranging from 0.25 to 0.60 pg/cell). These findings suggest that alginate production by rhizoplane-associated bacteria is enhanced by osmotic stress at all irrigation levels, as evidenced by higher median values compared to the respective controls (Figure 5C, orange boxplot).

In the reproductive phase and under control conditions, the percentage of alginate-producing rhizoplane bacteria (93% and 90%) was similar between 100% and 50% irrigation. However, a reduction (66%) was observed under 0% irrigation (Figure 5D, green donut charts). Although median alginate production is similar for all irrigation levels, the highest alginate producer (0.51 pg/cell) was isolated from roots exposed to 0% irrigation (Figure 5D, green boxplot). When the isolates were exposed to osmotic stress, the percentage of alginate-producing isolates increased progressively with decreasing irrigation (83%, 88%, and 96% for 100%, 50%, and 0% irrigation, respectively) (Figure 5D, orange donut charts). Alginate production levels also rose under osmotic stress relative to control (0% PEG) across all irrigation levels (Figure 5D, orange boxplot). Under osmotic stress, one strain isolated under 0% irrigation evidenced a remarkable ability to produce alginate (44.93 pg/cell).

Compared to the vegetative phase, alginate production by the bacterial community increased under control and stress conditions in the reproductive phase. Bacteria isolated from the rhizoplane under stress produced more alginate than those collected from the endosphere.

### 3.5. Production of Indole-3-Acetic Acid (IAA)

#### 3.5.1. Production of IAA by the Endophytic Bacteria

**Figure 6:**
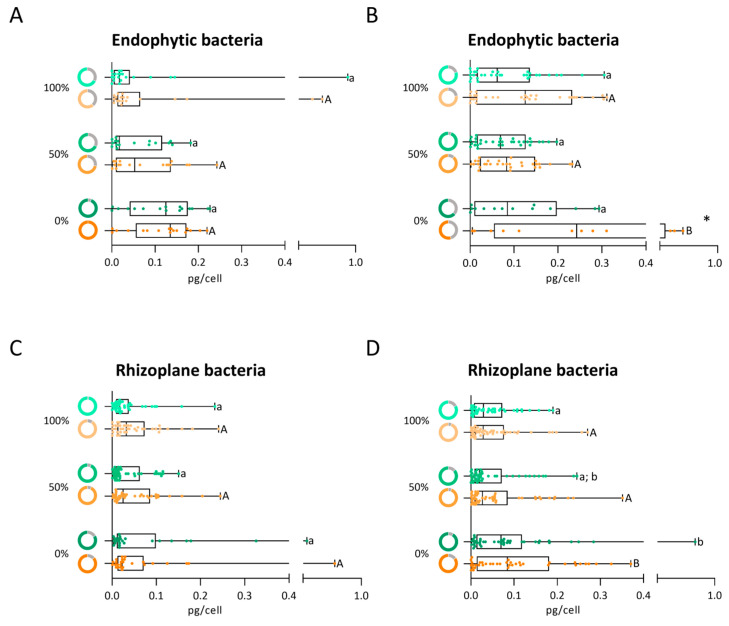
Indole-3-acetic acid (IAA) production by the bacterial strains in nanograms of IAA per cell (ng/cell). Control (green gradient) and osmotic stress (15% polyethylene glycol (PEG) 6000) (orange gradient) by bacteria isolated from the inside (endophytic) and outside (rhizoplane) of maize roots in two growth phases (vegetative and reproductive) exposed to three levels of irrigation (100%, 50%, and 0%). The median of the distribution is indicated by a black line, where the box represents positive values of the data. (**A**) Endophytic bacteria from plants in the vegetative phase, (**B**) endophytic bacteria from plants in the reproductive phase, (**C**) rhizoplane bacteria from plants in the vegetative phase, and (**D**) rhizoplane bacteria from plants in the re-productive phase. The respective donut charts represent the percentage of bacteria that are not capable (gray) or capable (green or orange) of IAA production. Boxplots include bacteria capable of producing IAA. Values are the means of three to six replicates ± standard error. Significant differences among levels of irrigation for control (0% PEG 6000) conditions are indicated with different lowercase letters, while for osmotic stress conditions (15% PEG 6000), they are indicated with different uppercase letters. Significant differences between osmotically stressed (15% PEG 6000) and non-stressed (0% PEG 6000) bacterial communities for each level of irrigation (100%, 50%, and 0%) are indicated by asterisks (*p* < 0.05).

Under control conditions, the percentage of endophytic bacteria from root maize in the vegetative phase capable of producing IAA was 68% for both 100% and 50% irrigation levels (Figure 6A, green donut charts). In contrast, a higher percentage of IAA-producing endophytes (94%) was observed in the bacterial community under the 0% irrigation treatment. Despite the median IAA production under 0% irrigation being higher than that under 100% and 50% irrigation, no statistically significant differences among irrigation levels were observed (Figure 6A, green boxplot). The isolate with the highest IAA production (0.92 pg/cell) was isolated from roots exposed to 100% irrigation. At osmotic stress, the percentage of IAA-producing isolates increased as irrigation decreased, with 61%, 72%, and 100% of isolates producing IAA under 100%, 50%, and 0% irrigation, respectively (Figure 6A, orange donut charts). Correspondingly, the median IAA production also increased with decreasing irrigation levels (Figure 6A, orange boxplot), although not statistically significant between conditions. Interestingly, the highest IAA-producing strains under stress (0.55 and 0.65 pg/cell) were isolated from the 100% irrigation endophytic community. Overall, an increase in median IAA production under stress was observed across all irrigation levels, indicating that osmotic stress enhances the auxin-producing capacity of endophytic bacteria (Figure 6A, orange boxplot).

In the reproductive phase, the irrigation levels (100%, 50%, and 0%) influenced the ability of endophytic bacteria to produce alginate (80%, 94%, and 64%, respectively) (Figure 6B, green donut charts). However, no statistically significant differences in median alginate production were observed among the irrigation levels (Figure 6B, green boxplot). The highest production (0.26 to 0.31 pg/cell) was exhibited by strains isolated under 0% and 100% irrigation conditions. Exposure to osmotic stress induced differences in the number of IAA-producing strains (78%, 89%, and 55%) among irrigation levels (100%, 50%, and 0%, respectively) (Figure 6B, orange donut charts). Although fewer IAA-producing strains were isolated under the 0% irrigation treatment, this community exhibited a significantly higher IAA production capacity than those at 100% and 50% irrigation levels (Figure 6B, orange boxplot). Despite the lower percentage, three strains isolated from 0% irrigation produced the highest individual level of IAA, between 0.51 and 0.64 pg/cell, under osmotic stress.

Overall, endophytic bacteria isolated from maize roots at varying irrigation levels in the reproductive phase, under both control and osmotic stress conditions, exhibited increased median IAA production in response to water stress when compared to the vegetative phase.

#### 3.5.2. Production of IAA by the Rhizoplane Bacteria

In the vegetative phase and non-stress conditions, a decrease in the number of rhizoplane bacteria able to produce IAA (98%, 93%, and 83%) was observed with the decrease in irrigation (Figure 6C, green donut charts). Nevertheless, the two highest IAA-producing strains (0.32 and 0.44 pg/cell) were isolated under 0% irrigation (Figure 6C, green boxplot). When the rhizoplane isolates were exposed to osmotic stress, the percentage of IAA-producing strains was similar among irrigation levels (91%, 93%, and 87% for 100%, 50% and 0% irrigation, respectively) (Figure 6C, orange donut charts). The highest IAA-producing strain under stress (0.73 pg/cell) was isolated from the 0% irrigation treatment (Figure 6C, orange boxplot). Overall, osmotic stress stimulated the ability of rhizoplane strains to produce IAA (Figure 6C, donut chart and boxplot).

Under control conditions, the percentage of rhizosphere bacteria from root maize in the reproductive phase capable of producing IAA is similar across all irrigation levels (83%, 87%, and 91% for 100%, 50%, and 0% irrigation, respectively) (Figure 6D, green donut charts). The strains from the 0% irrigation treatments exhibited higher median IAA production, statistically significant compared to the 100% irrigation treatment. (Figure 6D, green boxplot). The highest IAA production (0.79 pg/cell) was isolated from the 0% irrigation rhizoplane community. Exposure to osmotic stress changed the percentage of IAA-producing strains: 94% under both 100% and 50% irrigation, and 85% under 0% irrigation (Figure 6D, orange donut charts). The strains under 0% irrigation showed significant increases in IAA production compared to those under 100% and 50% irrigation treatments (Figure 6D, orange boxplot). The rhizoplane strain with highest IAA production (0.37 pg/cell) was isolated from the 0% irrigation treatment. Under osmotic stress, the number of strains producing IAA increased 11% and 7% under 100% and 50% irrigation, respectively (Figure 6D, donut charts). The median values show an increase in the amount of IAA produced with the decrease in water irrigation, enhanced with the exposure of the rhizoplane bacteria to osmotic stress (15% PEG).

Overall, rhizoplane bacteria isolated from maize roots at varying irrigation levels in the reproductive phase exhibited higher median IAA production under osmotic stress when compared to the vegetative phase.

### 3.6. Siderophore Production

#### 3.6.1. Siderophore Production by the Endophytic Bacteria

**Figure 7:**
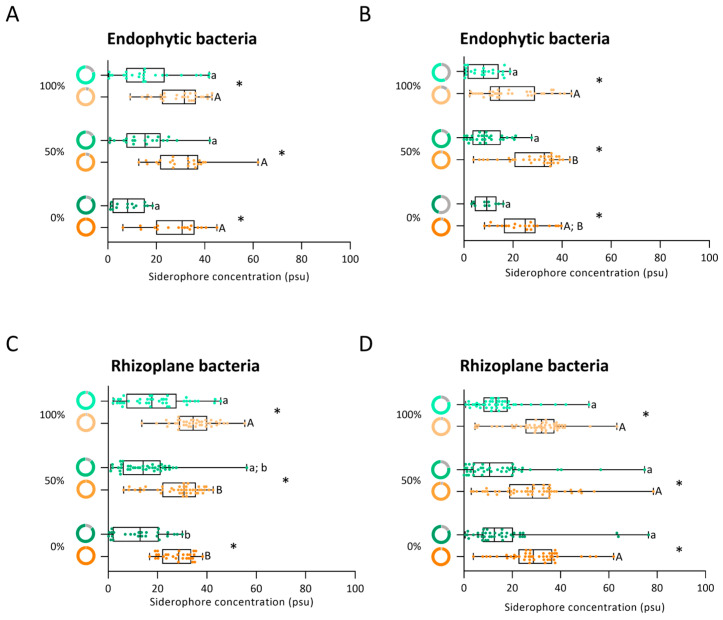
Siderophore production by the bacterial strains in percent siderophore units (*psu*). Control (green gradient) and osmotic stress (15% polyethylene glycol (PEG) 6000) (orange gradient) by bacteria isolated from the inside (endophytic) and outside (rhizoplane) of maize roots in two growth phases (vegetative and reproductive) exposed to three levels of irrigation (100%, 50%, and 0%). The median of the distribution is indicated by a black line, where the box represents positive values of the data. (**A**) Endophytic bacteria from plants in the vegetative phase, (**B**) endophytic bacteria from plants in the reproductive phase, (**C**) rhizoplane bacteria from plants in the vegetative phase, and (**D**) rhizoplane bacteria from plants in the reproductive phase. The respective donut charts represent the percentage of bacteria that are not capable (gray) or capable (green or orange) of siderophore production. Boxplots show bacteria capable of producing siderophores expressed in percent of siderophore units (*psu*). Values are the means of three to six replicates ± standard error. Significant differences among levels of irrigation for control (0% PEG 6000) conditions are indicated with different lowercase letters, while for osmotic stress conditions (15% PEG 6000), they are indicated with different uppercase letters. Significant differences between osmotically stressed (15% PEG 6000) and non-stressed (0% PEG 6000) bacterial communities for each level of irrigation (100%, 50%, and 0%) are indicated by asterisks (*p* < 0.05).

Irrigation levels (100%, 50%, and 0%) influenced the ability of bacteria to produce siderophores (81%, 84%, and 88%, respectively) in the root endosphere (Figure 7A, green donut charts). The decrease in the median siderophore production under 0% irrigation compared to the other irrigation conditions was not statistically significant (Figure 7A, green boxplot). The strains with higher ability to produce siderophores (20 to 42 *psu*) were isolated under 50% and 100% irrigation. Exposure to osmotic stress increased the number of strains able to produce siderophores across all irrigation treatments, with 94%, 92%, and 100% of strains producing siderophores under 100%, 50%, and 0% irrigation, respectively (Figure 7A, orange donut charts). Siderophore production levels also rose under osmotic stress relative to the control (0% PEG) across all irrigation levels (Figure 7A, brown donut charts). Despite all strains isolated under 0% irrigation producing siderophores, the strain exhibiting the highest production of siderophores (62 *psu*) was isolated under 50% irrigation. Overall, increased siderophore production by bacteria at all irrigation levels was observed under osmotic stress relative to control conditions (0% PEG).

In the reproductive phase, the irrigation levels (100%, 50%, and 0%) influenced the ability of endophytic bacteria to produce siderophores (58%, 86%, and 45%, respectively) (Figure 7B, green donut charts). Although median siderophore production slightly improved with decreasing irrigations levels, no statistically significant differences were observed among irrigation levels (Figure 7B, green boxplot). The strains evidencing the highest siderophore production (20 to 28 *psu*) were isolated from the 50% irrigation endophytic community. In the presence of osmotic stress (15% PEG), the percentage of strains able to produce siderophores was lower under 100% irrigation (88%) compared to 50% and 0% irrigation (97% and 95%, respectively) (Figure 7B, orange donut charts). Although median siderophore production was higher (<20 *psu*) in the 50% and 0% irrigation communities, only the 50% irrigation community was significantly different from the 100% irrigation community (Figure 7B, orange boxplot). The strains with the highest siderophore production were isolated from 100% and 50% irrigation treatments (44 and 43 *psu*, respectively). The endophytic bacterial community at all irrigation levels increased siderophore production under osmotic stress.

In general, the proportion of siderophore production strains remained relatively stable across the maize life cycle (the vegetative and reproductive phases) under the control condition (0% PEG). The strains improved siderophore production under osmotic stress (15% PEG). These findings suggest that siderophore production by endophytic bacteria is enhanced by osmotic stress at all irrigation levels in both phases, as evidenced by higher median values compared to the respective controls.

#### 3.6.2. Siderophore Production by the Rhizoplane Bacteria

In the vegetative phase, the percentage of siderophore-producing rhizoplane strains decreased as water irrigation reduced (95%, 89%, and 87% for 100%, 50%, and 0% irrigation, respectively) (Figure 7C, green donut charts), while median siderophore production indicated a significant decrease under 50% and 0% irrigation compared to 100% irrigation (Figure 7C, green boxplot). The highest siderophore producer (56 *psu*) was isolated from the 50% irrigation treatment. Exposure to osmotic stress (15% PEG) increased the percentage of strains able to produce siderophores (98%, 100%, and 100%), under all irrigation conditions (100%, 50%, and 0%, respectively) (Figure 7C, orange donut charts). Despite the increased percentage, median siderophore production reduced as irrigation decreased (Figure 7C, orange boxplot). The strain with the highest siderophore production (55 *psu*) was isolated under 100% irrigation.

In the reproductive phase, under control conditions, the irrigation levels did not influence the proportion of rhizoplane bacteria capable of producing siderophores (between 79% and 83%) (Figure 7D, green donut charts). The highest siderophore producer (76 *psu*) was isolated from the 0% irrigation treatment (Figure 7D, green boxplot). Exposure to osmotic stress (15% PEG) increased the number of strains able to produce siderophores, but the percentages remained similar between irrigation levels (96% to 98%) (Figure 7D, orange donut charts). No statistically significant differences in the median were found among irrigation levels (Figure 7D, orange boxplot). The strain with the highest siderophore production (78 *psu*) was isolated under 50% irrigation.

Overall, the exposure of strains to PEG-induced stress increased the siderophore production of bacteria isolated at all irrigation levels. However, siderophore production by the bacterial communities was stable throughout the maize life cycle regardless of irrigation levels of isolates.

### 3.7. Phosphate Solubilization

#### 3.7.1. Phosphate Solubilization by the Endophytic Bacteria

**Figure 8:**
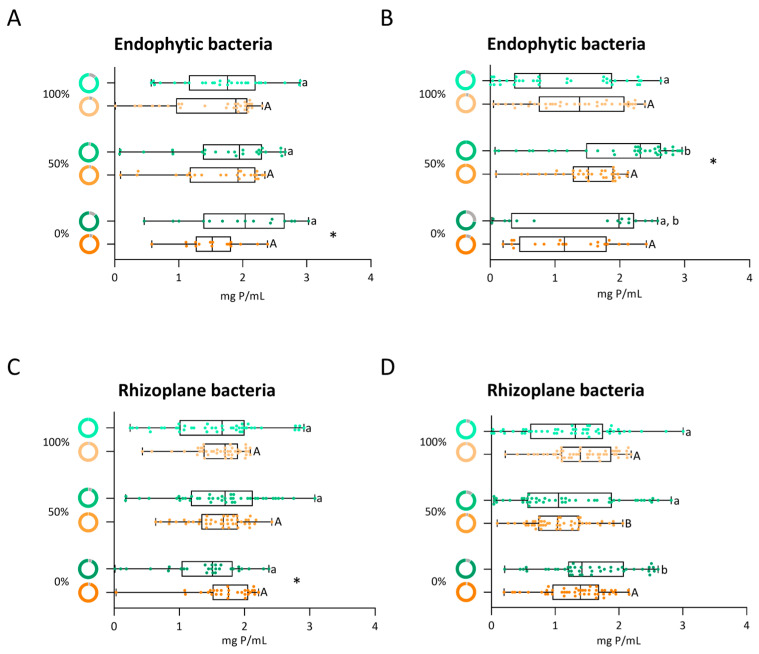
Phosphate solubilization by the bacterial strains in milligrams of phosphate per milliliter (mg P/mL). Control (green gradient) and osmotic stress (15% polyethylene glycol (PEG) 6000) (orange gradient) by bacteria isolated from the inside (endophytic) and outside (rhizoplane) of maize roots in two growth phases (vegetative and reproductive) exposed to three levels of irrigation (100%, 50%, and 0%). The median of the distribution is indicated by a black line, where the box represents positive values of the data. (**A**) Endophytic bacteria from plants in the vegetative phase, (**B**) endophytic bacteria from plants in the reproductive phase, (**C**) rhizoplane bacteria from plants in the vegetative phase, and (**D**) rhizoplane bacteria from plants in the reproductive phase. The respective donut charts represent the percentage of bacteria that are not capable (gray) or capable (green or orange) of phosphate solubilization. Boxplots include bacteria capable of solubilizing phosphate. Values are the means of three to six replicates ± standard error. Significant differences among levels of irrigation for control (0% PEG 6000) conditions are indicated with different lowercase letters, while for osmotic stress conditions (15% PEG 6000), they are indicated with different uppercase letters. Significant differences between osmotically stressed (15% PEG 6000) and non-stressed (0% PEG 6000) bacterial communities for each level of irrigation (100%, 50%, and 0%) are indicated by asterisks (*p* < 0.05).

Under control conditions, the percentage of endophytic bacteria from root maize during the vegetative phase capable of solubilizing phosphate was not significantly affected by the irrigation levels (87%, 96%, and 88% for 100%, 50%, and 0%, respectively) (Figure 8A, green donut charts). Median phosphate solubilization increased in the bacterial communities with decreasing irrigation treatments (Figure 8A, green boxplot). Furthermore, in the 100% and 0% irrigation treatments, bacterial strains exhibited the highest capacity to solubilize phosphate, ranging from 2.8 to 3.0 mg P/mL. When the same community was exposed to osmotic stress, differences in irrigation levels did not affect the proportion of phosphate-solubilizing strains (between 94% and 96%) (Figure 8A, orange donut charts). Nevertheless, under osmotic stress, phosphate solubilization (median) was lower in isolates under 0% irrigation than in those under 100% and 50% irrigation (Figure 8A, orange boxplot) and also lower compared to the control (0% PEG) (Figure 8A, boxplot). Interestingly, the strains exhibiting higher P solubilization abilities (2.5 to 3.0 mg P/mL) were observed under 15% PEG and isolated from all irrigation treatments. Upon exposure to osmotic stress, the 100% and 0% treatments exhibited an increased percentage of phosphate-solubilizing endophytes compared to the control (0% PEG) (Figure 8A, donut charts).

In the reproductive phase, the irrigation level appeared to influence the percentage of phosphate-solubilizing endophytic strains (88%, 100%, and 73% for 100%, 50%, and 0%, respectively) (Figure 8B, green donut charts). Median phosphate solubilization increased with decreasing irrigation level, and a significant difference was observed between 100% and 50% irrigation (Figure 8B, green boxplot). The higher phosphate solubilization ranged from 2.7 to 2.96 mg P/mL in strains isolated under 50% irrigation treatment. Exposure to 15% PEG reduced the difference in the percentage of phosphate-solubilizing strains among irrigation levels (95%, 100%, and 91% for 100%, 50%, and 0%, respectively) (Figure 8B, orange donut charts). The median phosphate solubilization was lower in strains isolated under 0% irrigation than under 100% and 50% irrigation (Figure 8B, orange boxplot). The two isolates with highest phosphate solubilization (2.2 and 2.4 mg P/mL) under osmotic stress were isolated under 100% and 0% irrigation, respectively.

In the presence of osmotic stress, the bacterial strains increased the percentage of phosphate-solubilizing strains across all irrigation regimes (Figure 8B, donut charts). However, a decrease in phosphate solubilization was observed in strains isolated under 50% and 0% irrigation, compared to control conditions (0% PEG) (Figure 8B, boxplot).

#### 3.7.2. Phosphate Solubilization by the Rhizoplane Bacteria

In the vegetative phase and under control conditions, the irrigation levels (100%, 50%, and 0%) did not significantly affect the percentage of phosphate-solubilizing rhizoplane bacteria (100%, 93%, and 93%, respectively) (Figure 8C, green donut charts). A small decrease in the median solubilization of phosphate in the bacterial community at 0% irrigation in comparison with the other levels of irrigation was observed (Figure 8C, green boxplot). Although all isolates under 100% irrigation treatments were able to solubilize phosphate, the rhizoplane bacteria with the highest phosphate solubilization (3.1 mg P/mL) were isolated under 50% irrigation. When the bacteria were exposed to osmotic stress, the irrigation levels (100%, 50%, and 0%) did not influence the percentage of phosphate-solubilizing rhizoplane strains (98%, 100%, and 97%, respectively) (Figure 8C, orange donut charts). The strain with the highest phosphate solubilization (2.4 mg P/mL) was isolated from roots exposed to 50% irrigation (Figure 8C, orange boxplot). In contrast, a higher proportion of phosphate solubilized was observed in the bacterial community with 0% irrigation relative to controls without osmotic stress (Figure 8C, boxplot).

In the reproductive phase, the irrigation levels did not influence the percentage of phosphate-solubilizing strains (between 88% and 91%) (Figure 8D, green donut charts). The phosphate-solubilizing rhizoplane bacteria with the highest values (2.7 to 3.0 mg P/mL) were isolated from 100% and 50% irrigation treatments (Figure 8D, green boxplot). Exposure to osmotic stress (15% PEG) kept the percentage of phosphate-solubilizing strains relatively stable across irrigation levels (between 96% and 98%) (Figure 8D, orange donut charts). Phosphate solubilization differed significantly between communities under 50% irrigation treatment and those under the other irrigation conditions (100% and 0%) (Figure 8D, orange boxplot). Interestingly, in the 100% irrigation treatment, five bacterial strains exhibited the highest phosphate solubilization, with values higher than 2.0 mg P/mL.

Overall, phosphate solubilization by rhizoplane bacteria increased under osmotic stress (Figure 8D, donut charts). Nevertheless, no discernible differences in median phosphate solubilization at 15% PEG were observed in strains from each irrigation level relative to the control (0% PEG).

## 4. Discussion

### 4.1. Marked Shifts in Maize-Associated Culturable Bacterial Communities Across Developmental Phases

#### 4.1.1. Isolation of Bacterial Community from Maize Root

An early study [64] found no significant variations in microbial community density during the maize life cycle; however, structural shifts occurred early on, stabilizing afterward. It was also observed that field site characteristics directly impact microbial density and structure in the maize rhizosphere [64]. These findings demonstrate the influence of soil composition on bacterial communities and their structure. A study that followed [38], indicated that the high bacterial density (CFU/g root fresh weight) in the soil at the beginning of maize vegetative phase (20 days) decreases and stabilizes quickly throughout the rest of the maize life cycle. Although plant development did not directly affect the total density of cultivable microflora, the presence of plants selectively influenced certain bacterial and fungal groups in the rhizosphere during the maize life cycle [38]. This demonstrates the impact of plants on soil microflora, likely due to root exudate production during plant growth [65]. A recent study demonstrated that the density (CFU/g soil) of the maize rhizosphere bacterial community declines during maize development, while minimum tillage and mulching practices enhance soil microbial biomass and bacterial diversity [66]. In contrast to these findings, our study demonstrated an increase in the bacterial density (CFU/ g soil) in the maize rhizosphere in the reproductive phase compared with the vegetative phase. Taking into consideration previous findings [67], the enhancement in rhizosphere microbial activity during the reproductive phase in our study is most likely due to changes in root exudation, root architecture, and plant–microbe interactions, instead of modifications associated with shifts in soil composition. Furthermore, we also verified that exposure to water stress increased microbial density compared to normal irrigation conditions. In a previous study, it was observed that some drought-tolerant rhizobia increased root colonization in the presence of drought and throughout the life cycle of maize [68]. The increased density observed in our study during plant development and presence of water stress could be related not only to the root exudates but also to the efficient root colonization by rhizobia, enhancing nutrient extraction, improving plant development, and boosting maize production under drought stress conditions [68].

Previous studies [69,70,71] using sequencing techniques have demonstrated a higher abundance and diversity of bacteria in the bulk soil and rhizosphere than in the root endosphere of maize plants. This was also observed in our study, where bacterial strain diversity was higher in the rhizoplane compared to the root endosphere. This may be related to the rhizoplane exposure to root exudates, which creates a nutrient-rich environment that supports a more favorable environment for diverse bacteria colonization [72]. On the other hand, the low diversity in endospheric bacteria could be related to the selective recruitment of bacterial species present in the bulk soil by maize seedlings [70,73].

Exposure to chilling conditions and nitrogen application can decrease and change the diversity and composition of micoibiomes in the rhizosphere and endosphere of maize [71,74]. A study by Tiziani et al. [75] observed that exposure to drought, heat, or a combination of both stresses led to modifications in root exudate composition, which in turn influenced the relative abundance of specific bacterial taxa in the maize rhizosphere. We also observed shifts in the bacterial community associated with maize roots in response to varying water availability.

In our study, we also observed phase-dependent changes in the relative abundance of cultivable bacteria associated with maize roots. Mougel et al. [76] also recorded differences in the genetic structure of microbial communities during plant development, as well as an increase in the level of symbiotic association during the transition between vegetative and reproductive stages. The variations in community structure associated with roots throughout the maize plant life cycle have been associated with exudate release by the root, as well as its growth [37].

Altogether, our study highlights how in maize root-associated bacterial communities, rhizoplane and endophytic bacteria are affected by plant growth phases and soil water availability. The distinct dynamics of these microbial communities across different soil moisture levels and developmental phases demonstrate the intricate interaction between environmental factors and plant biology. These insights provide important knowledge to enhance agricultural practices and deepen our understanding of plant–microorganism relationships.

#### 4.1.2. Bacterial Identification

##### Bacteria Isolated from the Vegetative vs. Reproductive Phase

Research analyzing the bacterial community associated with the maize rhizosphere has demonstrated that several factors, including the geographic location, soil type, maize variety, and plant’s developmental phase, can influence the interaction of the root with the microorganisms in the rhizosphere [25,37,38,39,77,78,79]. The importance of bacteria in the development of maize crops during the phase of high nutrient demand was demonstrated by Anzuay et al. [80], who noticed alterations in the structure of maize rhizobacterial community throughout the life cycle of maize plants, with considerable modifications occurring in the later phase of growth, increasing the genera of plant growth-promoting bacteria.

The plant developmental phase significantly influences microbial community composition independently of water availability. As plants progress through different growth stages, they undergo changes in physiology, metabolism, structure, and immune defenses, all of which shape microbial communities. For example, root exudate composition shifts from simple sugars and amino acids in seedlings to more complex secondary metabolites in mature plants, selectively enriching microbes adapted to these compounds [81]. Structural changes, such as root architecture or tissue differentiation, create new microhabitats that favor distinct microbial taxa, independent of moisture levels [82]. The reduction in root exudates or alterations in immune responses by the plant can also impact the microbial community [83]. This demonstrates that developmental stage influences microbial communities via nutrient availability, structural niches, and immune activity, independently of water availability.

In the present study, changes in the cultivable bacterial community over plant growth and development were observed for maize endophytic and rhizoplane communities. In irrigated soil during the vegetative phase (60 days), we observed a predominance of Pseudomonadaceae and Comamonadaceae families, with *Pseudomonas* and *Delftia* being the most abundant genera in endophytic community, while in the rhizoplane, a predominance of Pseudomonadaceae and Enterobacteriaceae families was observed, with *Pseudomonas* and *Enterobacter* being the most abundant genera. In the reproductive phase (102 days), there was a predominance of Enterobacteriaceae, Bacillaceae, and Pseudomonadaceae families, with the *Enterobacter*, *Priestia*, and *Pseudomonas* being the genera more present in the endophytic environment, while in the rhizoplane, there was a predominance of Pseudomonadaceae and Enterobacteriaceae families, with *Pseudomonas* and *Cedecea* being the most present genera. In contrast to our findings, the endosphere of maize roots planted in an experimental field in Merelbeke, Belgium, showed higher abundance of Proteobacteria, Oxalobacteraceae, Actinobacteria, and Streptomycetaceae [74]. This discrepancy was likely brought on by the study’s location and maize variety [37,79]. Our data allowed us to observe the dynamics of bacterial communities over the course of the maize life cycle, while the research in Merelbeke [74] was conducted during the early vegetative phase. However, Cavaglieri et al. [38] also noted that the bacterial community associated with maize roots shifted during the life cycle of maize, observing that after 60 days (vegetative phase), the genera *Bacillus*, *Arthrobacter*, *Listeria*, *Pseudomonas*, and *Agromyces* were predominant in the endosphere of maize roots, whereas the genera *Bacillus*, *Listeria*, *Pseudomonas*, and *Agromyces* were predominant in the rhizoplane [38]. During the maize reproductive phase (130 days), the most predominant genera in the endosphere were *Bacillus*, *Arthrobacter*, and *Azotobacter*, while in the rhizoplane, they were *Bacillus*, *Arthrobacter*, and *Azotobacter* [38]. Similarly to Cavaglieri et al. [38], throughout the vegetative phase of our study, *Pseudomonas* dominated the rhizoplane and endophitic bacterial communities. However, during the reproductive phase, the prevailing genera of the rhizoplane and endosphere differ. This could be connected to the characteristics of the agricultural soil, seasonal changes, or the maize variety [37,79,84].

A study conducted in different fields in Quebec analyzed the microbiota of the root from 20 different maize hybrids and observed the presence of *Pseudomonas*, *Bacillus*, and *Corynebacterium* genera in the endosphere and rhizoplane [78]. The genera *Serratia*, *Enterobacter*, *Acinetobacter*, *Klebsiella*, and *Agrobacterium* were only found in the rhizoplane, evidencing a lower number of genera present in the root endosphere compared to the rhizoplane [78]. In our study, we also observed that some bacterial taxa were exclusively isolated from the rhizoplane during the vegetative (*Agrobacterium* spp., *Paenarthrobacter* spp., members of *Lysobacteraceae* family, *Flavobacterium* spp., *Enterobacter* spp., *Acidovorax* spp., *Burkholderia* spp., and *Achromobacter* spp.) and the reproductive (*Acinetobacter* spp., *Cedeceas* spp., *Variovorax* spp., *Chitinophaga* spp., *Burkholderia* spp., *Bosea* spp., and *Achromobacter* spp.) phases. Conversely, some bacterial taxa were solely detected in the endophytic community during the vegetative phase, namely *Sphingomonas* spp., *Massilia* spp., *Herbaspirillum* spp., *Pantoea* spp., *Limnohabitans* spp. *Delftia* spp., and *Bosea* spp., while *Pseudoxanthomonas* spp., *Neorhizobiums* spp., *Mesorhizobium* spp., *Chiyseobacterium* spp., and *Brucella* spp. were exclusively found inside the roots in the reproductive phase. Although most endophytic bacteria originate from the root-surrounding soil and rhizosphere, plants continuously select beneficial microbes from this environment. This selective process shapes bacteria colonization within the root system, leading to the formation of a specialized endophytic community that enhances nutrient uptake, regulates growth, and modulates stress-related phytohormones [85].

Wang et al. [79] investigated the root endosphere and rhizosphere of 10 maize hybrids growing in a field trial in Brule, Nebraska, and found a decline in several bacterial families and genera in the endosphere (Gaiellaceae, Rhodoplanes, Sphingomonas, Micromonosporaceae, Alicyclobacillus, Oxalobacteraceae, and Asteroleplasma) and rhizosphere (Candidatus such as Nitrososphaera, Sinobacteraceae, and Oxalobacteraceae) during the later phases of the maize life cycle. On the other hand, in the same study, increases in other bacterial families and genera in both the root endosphere (Cytophagaceae, Mesorhizobium, Chitinophagaceae, Xanthomonadaceae, Myxocccales, *Pseudomonas*, *Burkholderia*, *Sphingobium*, Sphingobacteriaceae, Janthinobacteriaceae, Actinosynnemataceae, Amycolatopsis, *Chitinophaga*, Streptomycetaceae, and Comamonadaceae) and rhizosphere (*Kaistobacter*, Comamonadaceae, Xanthomonadaceae, *Arthrobacter*, Chitinophagaceae, Sphingobacteriaceae, Oxalobacteraceae, Sphingomonadaceae, *Bradyrhizobium*, *Mesorhizobium*, Streptomycetaceae, and *Sphingobium*), were observed during maize plants development [79]. However, certain taxa like *Bradyrhizobium*, *Mesorhizobium*, *Pseudomonas*, and Rhodospirollaceae showed no significant changes in the rhizosphere throughout the maize life cycle [79]. Similarly in our study, a decrease in almost all bacterial genera, except for *Pantoea* and *Priestia*, was observed in the endophytic community through the maize life cycle. This generalized decline in endophytic bacterial genera present in the vegetative phase could be related to the emergence of other genera, such as *Neorhizobium*, *Agrobacterium*, *Mesorhizobium*, *Arthrobacter*, *Microbacterium*, *Chiyseobacterium*, *Enterobacter*, *Paraburkholderia*, *Brucella*, *Bradyrhizobium*, and *Neobacillus*, during the reproductive phase. Regarding the rhizoplane community, we also observed a decline in almost all bacterial genera, except for *Agrobacterium* and *Cedecea*, and the emergence of other genera (*Pseudoxanthomonas*, *Acinetobacter*, *Paenarthrobacter*, *Arthrobacter*, *Pantoea*, *Variovorax*, *Chitinophaga*, *Paraburkholderia*, and *Bosea*) in the reproductive phase. The observed variations in the endophytic and rhizospheric bacterial communities throughout the maize life cycle suggest that the plant developmental phase significantly influences the composition and dynamics of soil bacterial populations. These changes may result from dynamic interactions between plant physiology, changes in root exudation profiles, microbial succession, and environmental conditions. Throughout development, maize actively modulates its microbiome to align with changing nutritional requirements and to better respond to the prevailing conditions [86].

The overall increase in bacterial diversity observed during the reproductive phase of maize compared to the vegetative stage can be attributed to several physiological and ecological factors associated with plant development. As maize plants mature, they undergo significant changes in root exudation patterns, tissue composition, and microhabitat availability, which together create a wider range of ecological niches for microbial colonization [87]. In a study on rice plants, the more complex microbial communities observed during the reproductive phase compared with earlier stages were attributed to a stronger selective pressure exerted by the plant on the rhizosphere microbiome at this stage [88]. Previous studies have shown that exudation rates decline as maize plants develop; however, despite this decrease, the proportion of soluble carbohydrates in root exudates increases during the reproductive phase compared to other exudate compounds [89,90]. These exudates serve as a carbon source for diverse microbial taxa, supporting the establishment of both generalist and specialized bacteria [91].

##### Osmotic Stress Alters the Composition of Bacterial Communities Associated with Maize Roots

Maize is generally considered tolerant to water stress in the vegetative phase but becomes highly sensitive during the periods of tasseling, silking, and pollination, with moderate sensitivity during the grain-filling phase [92]. The stress-induced alterations observed in the bacterial structure of cultivable root-associated bacterial communities in our study and also in the literature [93,94] may represent one of several factors contributing to the increased vulnerability of maize under field conditions. Water stress has been demonstrated to diminish the diversity of bacterial communities associated with maize roots [79] and to impact bacterial cell surface properties (wettability, chemical composition, and charge) [95], which may adversely affect the functionality of PGPB traits during periods of stress. In our study, in the vegetative phase, osmotic stress led to an increased relative abundance of the Enterobacteriaceae family in both the endosphere and rhizoplane of maize roots, suggesting that members of this family may possess adaptive mechanisms that confer tolerance to low water availability. In contrast, a decline in Pseudomonadaceae within the endophytic bacterial community and Rhizobiaceae both in endophytic and rhizoplane compartments indicates that certain taxa within these families may be more sensitive to osmotic stress. Additionally, members of the Microbacteriaceae family were identified in the root endosphere under both low- and no-irrigation conditions, further highlighting the shifts in microbial assemblages in response to water availability.

Changes were also observed in the reproductive phase under osmotic stress, with an increased relative abundance of Xanthomonadaceae in both the endophytic and rhizoplane bacterial community, along with Bacillaceae and Enterobacteriaceae in the rhizoplane, suggesting that members of these families can adapt to low water availability. Conversely, Pseudomonadaceae showed a notable decline in the endophytic bacterial community under severe drought (0% irrigation), as well as in the rhizoplane. Bacillaceae also declined within the endophytic compartment, indicating that not all members of drought-associated families uniformly tolerate osmotic stress across different root compartments.

The variations observed in the bacterial community during vegetative and reproductive phases under water stress may be related to the direct effects of drought on root and soil structure and composition [4]. The composition of root exudates varies according to plant genotype, environmental conditions, and biotic interactions. Water stress has also been shown to alter root exudation patterns in plants, which in turn modulates the rhizosphere microbial community [96]. From the vegetative to the reproductive phase under stress conditions, it was possible to observe a decrease in the Pseudomonadaceae family and an increase in the Enterobacteriaceae, Bacillaceae Flavobacteriaceae, Microbacteriaceae, Oxalobacteraceae, and Xanthomonadaceae families, demonstrating changes over time in the dominance of the Pseudomonadaceae family in the endophytic bacterial community exposed to water deficit, which allowed an increase in other families in the endosphere of maize roots. In the rhizoplane bacterial community, the decrease in Flavobacteriaceae and Pseudomonadaceae under water deficit may have facilitated the increased relative abundance of families such as Bacillaceae, Burkholderiaceae, Enterobacteriaceae, and Microbacteriaceae. These shifts suggest a possible competitive or functional replacement by taxa better adapted to low water availability. Previous studies have reported a reduction in *Pseudomonas* populations under both salinity [97] and water stress [98]. Members of this genus are Gram-negative, and the abundance of Gram-negative (*Proteobacteria*, *Verrucomicrobia* and *Bacteroidetes*) bacteria generally decreases under stress, with *Proteobacteria* being particularly drought-sensitive [99]. In contrast, Gram-positive bacteria (*Firmicutes*, *Actinobacteria* and *Acidobacteria*) often increase significantly under these conditions [99]. The increase in *Microbacterium*, a Gram-positive genus within the phylum Actinobacteria, may be attributed to its thick, hydrophobic cell walls, rich in mycolic acids, which have the ability to use inorganic nitrogen to produce extracellular enzymes that degrade complex organic compounds and degrade plant residues [99]. The increase in *Enterobacter* under water stress could be related to their tolerance to osmotic stress, production of plant growth-promoting bioactive compounds, and capacity to metabolize different carbon sources [100,101,102]. These physiological and structural adaptations confer a competitive advantage to drought-tolerant taxa, resulting in the observed decline in *Pseudomonas* and concomitant enrichment of *Enterobacter* and *Mycobacterium* populations in water-stressed soils.

Wang et al. [79] observed that water stress significantly altered rhizobacterial communities associated with maize roots in a field trial. In the maize root endosphere, they observed an increase in genera such as *Amycoltopsis* and *Acinetobacter*, alongside a reduction in families like Cytophagaceae and Chitinophagaceae and the order Myxococcales. In the rhizosphere, these authors observed that water stress was associated with an increased abundance of *Kaistobacter*, Oxalobacteraceae, *Bradyrhizobium*, Streptomycetaceae, and *Sphingobium*, while genera such as *Arthrobacter* and *Candidatus Nitrososphaera* decreased [79]. In our study changes in communities exposed to water stress were also observed; however, most genera and families that underwent alterations do not align with the findings of the study of Wang et al. [79], probably because of the different conditions (maize variety and growth conditions).

Alterations in the root-associated bacterial community of maize are not only influenced by stress such as drought, cold, and high temperature in the soil, they are also influenced by plant-mediated responses to such stressful situations. When maize plants are exposed to stressful conditions such as drought, heat, or both, a change in the bacterial community associated with the roots is observed, which leads to an increase in bacterial groups associated with positive effects on plants [103]. Tiziani et al. [75], demonstrated that maize roots exposed to drought, heat, and their combination released distinct sets of exudates depending on the stress condition. These stress-specific exudates significantly altered the abundance of certain bacterial groups, some of which are known to confer beneficial effects on plant growth and stress resilience. This highlights the dynamic interplay between plant responses and root microbiome composition under abiotic stress.

### 4.2. Bacteria Exposure to Osmotic Stress

Water plays an important role in soil functions and is essential for the survival of plants and microorganisms. Reduced soil moisture decreases water availability, which in turn limits substrate diffusion and ultimately reduces the accessibility of nutrients for microbial communities [104]. Gram-positive bacteria are generally more tolerant to increased osmotic potential than Gram-negative bacteria, owing to differences in cell wall structure and stress response mechanisms [105].

Our data indicate that bacteria isolated from the endophytic and rhizoplane communities of maize roots under osmotic stress are more tolerant to water deficit. This finding aligns with previous studies showing that water potential significantly shapes the microbial community structure and composition, often resulting in decreased microbial biomass, respiration, and metabolic activity [106,107]. These changes are attributed to selective pressures that favor microorganisms that can tolerate low water potential [106,107].

In our study, the percentage of osmotic stress-tolerant bacteria increased over time, particularly during the reproductive phase of maize, which could imply bacterial community adaptation to prolonged stress exposure. Similar patterns were reported by Pereira et al. [108], who observed that microbial communities initially responded negatively to drought stress, but over time, community function improved, with increased metabolism, mobility, and secretion activity, especially in some genera.

### 4.3. Plant Growth Promotion of Bacteria When Exposed to Osmotic Stress

Plant growth-promoting bacteria are important not only to aid plant development but also to minimize the effects of stress [106,109]. Different studies have discussed the characteristics and benefits of these bacteria; however, to the best of our knowledge, this is the first study to observe these characteristics under normal and osmotic stress conditions in a microbial community isolated from maize roots.

Studies examining individual bacterial taxa demonstrated that stress-induced responses can differ markedly between strains, suggesting that adaptation to stress is more often strain-specific than species-specific. Bandeppa et al. [110] observed that two isolates belonging to *Bacillus* in the presence of osmotic stress (20% PEG) increased IAA and exopolysaccharide production, but did not observe significant changes in phosphate (P) solubilization. In other studies, a decrease in IAA production was observed in five *Pseudomonas* sp. [111] and *Bacillus* sp. and *Staphylococcus* sp. [112] in the presence of osmotic stress (25% PEG [111] and 11% to 32% PEG [112], respectively). The exposure of five *Pseudomonas* sp. isolates to osmotic stress (25% PEG) led to a decrease in P solubilization, but siderophore production was maintained [111]. It should be noted that different plant growth-promoting bacteria can have different effects depending on the maize variety [113].

In our study, analysis of the three cultivable bacterial communities (100%, 50%, and 0% irrigation levels) isolated during the vegetative phase showed that the community exposed to 0% irrigation possesses a higher proportion of alginate-producing strains either from endophytic or rhizoplane compartments. While alginate production in the endophytic bacterial community was most prominent under 100% and 50% irrigation, the rhizoplane community associated with 0% irrigation displayed the highest percentage of alginate producers. Upon exposure to osmotic stress, both vegetative and reproductive phase-derived communities demonstrated an overall increase in alginate production, with the most pronounced response observed in rhizoplane communities originating from the 0% irrigation treatment. This finding aligns with other studies that related osmotic stress (20% PEG) with stimulation of alginate production across various bacterial genera [114]. In our study, the observed increase in alginate production among all three bacterial communities under osmotic stress likely reflects a microbial adaptive strategy to mitigate water stress, confirming a previous study of our laboratory that relates bacterial osmotolerance with the ability to produce alginate [114]. Alginate, an exopolysaccharide, enhances bacterial survival, promotes biofilm formation and water retention around roots, and stabilizes the rhizosphere under water stress [115], enhancing microbial survival under desiccation. Chang et al. [116] showed that when *Pseudomonas* were exposed to water deficit, there was an increase in alginate production, modifying the structure of the biofilm and allowing better retention of water. In our study, bacteria isolated from the rhizoplane under 0% irrigation evidenced a superior ability to produce alginate, which likely enhances their survival in water-deficit environments. This trait contributes to plant drought resilience by promoting water retention in the rhizosphere, thus maintaining moisture near the root surface. Liu et al. [117] inferred that plant drought tolerance may be enhanced by alginate oligosaccharides through interactions with ABA-dependent signaling pathways. These findings underscore the capacity of bacterial communities to adapt to severe osmotic stress by increasing alginate production, particularly during the maize reproductive phase, when improving root-associated conditions is critical for plant development.

In our study, the endophytic bacterial community isolated during the vegetative phase under severe stress (0% irrigation) exhibited the highest levels of IAA, both under control and osmotic stress conditions. In the reproductive phase, IAA production increased across all three communities compared to the vegetative phase. The highest IAA-producing strains were consistently found in the endophytic and rhizoplane communities of maize roots exposed to 0% irrigation, with enhanced production observed under osmotic stress. This pattern suggests a functional adaptation of the microbial community to support the physiological needs of maize under drought conditions. An increase in IAA enables plants to allocate more carbohydrates to their roots, enhancing root growth [118]. Increased root growth under stress conditions increases the plant’s capacity to access water and nutrients under limiting conditions. IAA, an essential auxin produced by bacteria, affects root structure and abiotic stress tolerance, enhancing plant growth, root elongation, water absorption, and climate-resilient agriculture through improved crop performance [119]. Regarding siderophore production, our results revealed only modest differences among bacterial communities. Sandhya et al. [111] showed that siderophore production remained stable under osmotic stress conditions in five bacterial strains; however, a small decrease in IAA and phosphate solubilization was also observed. Nevertheless, some *Streptomyces* were found to increase siderophore production in response to osmotic stress induced by NaCl [120]. In our study, the production of siderophores generally increased under stress conditions, particularly in bacteria isolated from the rhizoplane. This trend was maintained during the reproductive phase. The increase in siderophore production makes iron (Fe) more available in the soil, helping plants develop under stress conditions [121]. As a cofactor in important metalloenzymes like catalase, superoxide dismutase, and cytochromes and electron transport chains, Fe is necessary for plants and supports enzymatic processes that increase drought tolerance [122].

Phosphate solubilization capacity was also evaluated. Although across all conditions, phosphate solubilization was observed in a greater number of isolates under osmotic stress, the total amount of solubilized phosphate decreased in endophytic strains exposed to lower soil irrigation. Siddique et al. [123] observed that maize endophytic bacterial isolates capable of surviving under different osmotic stress conditions (10, 20, and 30% PEG 6000) had the ability to solubilize phosphate. In a study conducted by Kour et al. [124], 193 bacterial strains were isolated from the rhizosphere, and 167 were able to solubilize phosphate. However, among the 51 isolates tolerant to osmotic stress (5% PEG 8000), only 20 continued to exhibit the ability to solubilize phosphate under stress conditions. Our results revealed that rhizoplane bacteria isolated from both vegetative and reproductive phases evidenced a general decline in P solubilization under osmotic stress. Bacteria isolated from the vegetative phase at 0% irrigation evidenced a different pattern, with a significant increase in phosphate solubilization capacity when exposed to osmotic stress, suggesting a unique adaptation of this community to extreme drought conditions. We also observed a general decrease in the amount of P solubilization by the bacterial communities from the vegetative to the reproductive phase. This is consistent with findings by Vardharajula et al. [125], who observed that of 10 *Bacillus* strains tolerant to osmotic stress (−0.73 MPa), seven could solubilize phosphate (20–61 mg/mL). However, under stress conditions, their solubilization capacity decreased to 10–42 mg/mL. Similarly, other studies [126,127] have also observed that as osmotic stress increases (5–8% PEG 8000), the number of bacteria capable of solubilizing phosphate declines.

P-solubilizing bacteria are important for increasing available phosphorus and thus support maize growth. Adnan et al. [128] observed that inoculating maize with phosphate-solubilizing bacteria increased the development of plants mainly when the soil was supplemented with a source of P. Osmotolerant bacteria able to solubilize P may further benefit plants grown under water-deficit conditions by increasing the accumulation of osmolytes and decreasing oxidative damage [127]. Additionally, these bacteria can stimulate maize growth and uptake of nitrogen (N), phosphorus (P), and potassium (K), but these characteristics can change with soil composition [129]. In nutrient-deficient soils, the effects of bacteria on plant growth are more evident [129]. The application of microbial consortia, rather than single-strain inoculants, has been shown to be more effective for increasing plant development. Consortia offer a broader range of beneficial traits and functional redundancy, making microbial inoculants more resilient to environmental fluctuations. Therefore, microbial consortia are considered a promising and sustainable alternative to chemical fertilizers in agricultural systems [130].

## 5. Conclusions

This study demonstrated that the bacterial community associated with maize roots adapts to water-deficit conditions, resulting in compositional and functional shifts across the maize life cycle. A consistent decrease in *Pseudomonas* spp. within the endophytic compartment was observed under 0% irrigation across both vegetative and reproductive phases. Similarly, a decline in *Pseudomonas* spp. in the rhizoplane was noted over time, while drought stress led to an increase in *Enterobacter* spp., highlighting the influence of irrigation level on the composition of rhizoplane-associated bacterial communities.

Functional characterization related to nutrient availability and alginate production of the cultivable communities revealed that rhizoplane bacteria exhibited higher production of alginate and siderophores compared to endophytic bacteria, suggesting a key role of the rhizoplane microbiota in supporting plant resilience under stress. In contrast, elevated IAA production was a distinguishing feature of endophytic communities, though increases in IAA and alginate production were observed in rhizoplane isolates as well, particularly over time, indicating an adaptive response aligned with both environmental conditions and host plant developmental needs. During the reproductive phase, maize is particularly sensitive to drought, and the associated plant growth-promoting bacteria (PGPB) can respond by increasing alginate and IAA production.

Under osmotic stress (15% PEG 6000), all communities showed increased production of plant growth-promoting traits, except for phosphate solubilization, which generally decreased under stress. Notably, endophytic and rhizoplane communities isolated from maize under 0% irrigation continued to express high levels of alginate, IAA, and siderophore production under both control and stress conditions. Moreover, under extreme water stress, a greater number of isolates tolerant to osmotic stress were isolated from the rhizoplane during the reproductive stage. These findings underscore the value of isolating bacteria directly from highly stressed environments, as such isolates are more likely to retain their beneficial traits under similar challenging conditions. Consequently, these stress-adapted bacterial strains hold potential as bioinoculants for enhancing maize performance in water-limited agricultural systems.

## Data Availability

The raw data supporting the conclusions of this article will be made available by the corresponding author on request.

## Supplementary Materials

The following supporting information can be downloaded at: https://www.mdpi.com/article/10.3390/biology14121694/s1, Table S1: Statistical analysis of endophytic bacterial plant growth-promoting traits under control (0% of polyethylene glycol (PEG) 6000) and osmotic stress (15% of PEG 6000) conditions; Table S2: Statistical analysis of the rhizoplane bacterial plant growth-promoting (PGP) traits under control (0% of polyethylene glycol (PEG) 6000) and osmotic stress (15% of PEG 6000) conditions; Table S3: Statistical analysis of the colony-forming unit (CFU) data, based on the averages of technical replicates (Table S4); Table S4: Raw colony-forming unit (CFU) data; Table S5: GenBank accession numbers and identification at the genus/family level.

## Abbreviations

The following abbreviations are used in this manuscript:

CFUs	colony-forming units
DMB	dimethyl methylene blue
IAA	indole acetic acid
PEG	polyethylene glycol
PGPB	plant growth-promoting bacteria
YMA	yeast mannitol agar
YMB	yeast broth mannitol

## References

[1] Lee H., Romero J., IPCC, Core Writing Team (2023). Climate Change 2023: Synthesis Report. Contribution of Working Groups I, II and III to the Sixth Assessment Report of the Intergovernmental Panel on Climate Change.

[2] Toreti A., Bavera D., Acosta Navarro J., Arias-Muñoz C., Avanzi F., Marinho Ferreira Barbosa P., De Jager A., Di Ciollo C., Ferraris L., Fioravanti G. (2023). Drought in Europe March 2023: GDO Analytical Report.

[3] Siebielec S., Siebielec G., Klimkowicz-Pawlas A., Gałązka A., Grządziel J., Stuczyński T. (2020). Impact of Water Stress on Microbial Community and Activity in Sandy and Loamy Soils. Agronomy.

[4] Naylor D., Coleman-Derr D. (2018). Drought Stress and Root-Associated Bacterial Communities. Front. Plant Sci..

[5] Meng Q., Chen X., Lobell D.B., Cui Z., Zhang Y., Yang H., Zhang F. (2016). Growing Sensitivity of Maize to Water Scarcity under Climate Change. Sci. Rep..

[6] Ojuederie O., Olanrewaju O., Babalola O. (2019). Plant Growth Promoting Rhizobacterial Mitigation of Drought Stress in Crop Plants: Implications for Sustainable Agriculture. Agronomy.

[7] Ben Gaied R., Brígido C., Sbissi I., Tarhouni M. (2024). Sustainable Strategy to Boost Legumes Growth under Salinity and Drought Stress in Semi-Arid and Arid Regions. Soil Syst..

[8] Hoorman J.J. (2011). The Role of Soil Bacteria.

[9] Sahu N., Vasu D., Sahu A., Lal N., Singh S.K. (2017). Strength of Microbes in Nutrient Cycling: A Key to Soil Health. Agriculturally Important Microbes for Sustainable Agriculture.

[10] Benard P., Bickel S., Kaestner A., Lehmann P., Carminati A. (2023). Extracellular Polymeric Substances from Soil-Grown Bacteria Delay Evaporative Drying. Adv. Water Resour..

[11] Guo Y.-S., Furrer J.M., Kadilak A.L., Hinestroza H.F., Gage D.J., Cho Y.K., Shor L.M. (2018). Bacterial Extracellular Polymeric Substances Amplify Water Content Variability at the Pore Scale. Front. Environ. Sci..

[12] Harris R.F., Parr J.F., Gardner W.R., Elliott L.F. (1981). Effect of Water Potential on Microbial Growth and Activity. Water Potential Relations in Soil Microbiology.

[13] Tan S., Gu Y., Yang C., Dong Y., Mei X., Shen Q., Xu Y. (2016). *Bacillus amyloliquefaciens* T-5 May Prevent *Ralstonia solanacearum* Infection through Competitive Exclusion. Biol. Fertil. Soils.

[14] de Weert S., Bloemberg G.V. (2007). Rhizosphere Competence and the Role of Root Colonization in Biocontrol. Plant-Associated Bacteria.

[15] Weller D.M., Raaijmakers J.M., Gardener B.B.M., Thomashow L.S. (2002). Microbial Populations Responsible for Specific Soil Suppressiveness to Plant Pathogens. Annu. Rev. Phytopathol..

[16] Vessey J.K. (2003). Plant Growth Promoting Rhizobacteria as Biofertilizers. Plant Soil.

[17] Egamberdieva D., Shrivastava S., Varma A., Egamberdieva D., Shrivastava S., Varma A. (2015). Plant-Growth-Promoting Rhizobacteria (PGPR) and Medicinal Plants.

[18] Racioppo A., d’Amelio A., De Santis A., Bevilacqua A., Corbo M.R., Sinigaglia M. (2023). Potential Use of Plant Growth-Promoting Bacteria to Enhance Growth and Soil Fertility in Marginal Areas: Focus on the Apulia Region, Italy. Agronomy.

[19] Bonaterra A., Badosa E., Daranas N., Francés J., Roselló G., Montesinos E. (2022). Bacteria as Biological Control Agents of Plant Diseases. Microorganisms.

[20] Shiferaw B., Prasanna B.M., Hellin J., Bänziger M. (2011). Crops That Feed the World 6. Past Successes and Future Challenges to the Role Played by Maize in Global Food Security. Food Secur..

[21] OECD/FAO Cereals (2021). OECD-FAO Agricultural Outlook 2021–2030.

[22] McMillen M.S., Mahama A.A., Sibiya J., Lübberstedt T., Suza W.P. (2022). Improving Drought Tolerance in Maize: Tools and Techniques. Front. Genet..

[23] Cruz C., Cardoso P., Santos J., Matos D., Figueira E. (2022). Bioprospecting Soil Bacteria from Arid Zones to Increase Plant Tolerance to Drought: Growth and Biochemical Status of Maize Inoculated with Plant Growth-Promoting Bacteria Isolated from Sal Island, Cape Verde. Plants.

[24] Martínez-Viveros O., Jorquera M., Crowley D., Gajardo G., Mora M. (2010). Mechanisms and Practical Considerations Involved in Plant Growth Promotion by Rhizobacteria. J. Soil Sci. Plant Nutr..

[25] Cun H., Munir S., He P., Wu Y., He P., Ahmed A., Che H., Li J., He Y. (2022). Diversity of Root Endophytic Bacteria from Maize Seedling Involved in Biocontrol and Plant Growth Promotion. Egypt. J. Biol. Pest Control.

[26] Montañez A., Blanco A.R., Barlocco C., Beracochea M., Sicardi M. (2012). Characterization of Cultivable Putative Endophytic Plant Growth Promoting Bacteria Associated with Maize Cultivars (*Zea mays* L.) and Their Inoculation Effects in Vitro. Appl. Soil Ecol..

[27] Breedt G., Labuschagne N., Coutinho T.A. (2017). Seed Treatment with Selected Plant Growth-promoting Rhizobacteria Increases Maize Yield in the Field. Ann. Appl. Biol..

[28] Gholami A., Shahsavani S., Nezarat S. (2009). The Effect of Plant Growth Promoting Rhizobacteria (PGPR) on Germination, Seedling Growth and Yield of Maize. World Acad. Sci. Eng. Technol. Int. J. Agric. Biosyst. Eng..

[29] Jarak M., Mrkovački N., Bjelić D., Jošić D., Hajnal-Jafari T., Stamenov D. (2012). Effects of Plant Growth Promoting Rhizobacteria on Maize in Greenhouse and Field Trial. Afr. J. Microbiol. Res..

[30] Nezarat S., Gholami A. (2008). Screening Plant Growth Promoting Rhizobacteria for Improving Seed Germination, Seedling Growth and Yield of Maize. Pak. J. Biol. Sci..

[31] Ahmed A., Sultan T., Qadir G., Afzal O., Ahmed M., Shah S.-U.-S., Asif M., Ali S., Mehmood M.Z. (2020). Impact Assessment of Plant Growth Promoting Rhizobacteria on Growth and Nutrient Uptake of Maize (*Zea mays*). Pak. J. Agric. Res..

[32] Lin Y., Watts D.B., Kloepper J.W., Feng Y., Torbert H.A. (2020). Influence of Plant Growth-Promoting Rhizobacteria on Corn Growth under Drought Stress. Commun. Soil Sci. Plant Anal..

[33] Hafez E.M., Osman H.S., Gowayed S.M., Okasha S.A., Omara A.E.-D., Sami R., Abd El-Monem A.M., Abd El-Razek U.A. (2021). Minimizing the Adversely Impacts of Water Deficit and Soil Salinity on Maize Growth and Productivity in Response to the Application of Plant Growth-Promoting Rhizobacteria and Silica Nanoparticles. Agronomy.

[34] Silva I., Alves M., Malheiro C., Silva A.R.R., Loureiro S., Henriques I., González-Alcaraz M.N. (2022). Short-Term Responses of Soil Microbial Communities to Changes in Air Temperature, Soil Moisture and UV Radiation. Genes.

[35] Berendsen R.L., Pieterse C.M.J., Bakker P.A.H.M. (2012). The Rhizosphere Microbiome and Plant Health. Trends Plant Sci..

[36] Huang X.-F., Chaparro J.M., Reardon K.F., Zhang R., Shen Q., Vivanco J.M. (2014). Rhizosphere Interactions: Root Exudates, Microbes, and Microbial Communities. Botany.

[37] Bourceret A., Guan R., Dorau K., Mansfeldt T., Omidbakhshfard A., Medeiros D.B., Fernie A.R., Hofmann J., Sonnewald U., Mayer J. (2022). Maize Field Study Reveals Covaried Microbiota and Metabolic Changes in Roots over Plant Growth. MBio.

[38] Cavaglieri L., Orlando J., Etcheverry M. (2009). Rhizosphere Microbial Community Structure at Different Maize Plant Growth Stages and Root Locations. Microbiol. Res..

[39] Kong X., Han Z., Tai X., Jin D., Ai S., Zheng X., Bai Z. (2020). Maize (*Zea mays* L. Sp.) Varieties Significantly Influence Bacterial and Fungal Community in Bulk Soil, Rhizosphere Soil and Phyllosphere. FEMS Microbiol. Ecol..

[40] Gao Y., Zhao Y., Li P., Qi X. (2023). Responses of the Maize Rhizosphere Soil Environment to Drought-Flood Abrupt Alternation Stress. Front. Microbiol..

[41] Ibarra J.G., Colombo R.P., Godeas A.M., López N.I. (2020). Analysis of Soil Bacterial Communities Associated with Genetically Modi Fi Ed. Appl. Soil Ecol..

[42] Hartwig R.P., Santangeli M., Würsig H., Roldán M.M., Yim B., Lippold E., Tasca A., Oburger E., Tarkka M., Vetterlein D. (2025). Drought Response of the Maize Plant–Soil–Microbiome System Is Influenced by Plant Size and Presence of Root Hairs. Ann. Bot..

[43] Carter K.R., Nachtsheim A.C., Dickman L.T., Moore E.R., Negi S., Heneghan J.P., Sabella A.J., Steadman C.R., Albright M.B.N., Anderson C.M. (2023). Drought Conditioning of Rhizosphere Microbiome Influences Maize Water Use Traits. Plant Soil.

[44] Sá C., Figueira E., Cardoso P. (2025). Biochemical Response of Maize Plants Grown in the Field Under Different Water Availability: Evaluating the Influence of Leaf Position and Growth Stage. Agronomy.

[45] Somasegaran P., Hoben H.J. (1994). Handbook for Rhizobia: Methods in Legume-Rhizobium Technology. P. Somasegaran, H. J. Hoben. Q. Rev. Biol..

[46] Vincent J.M. (1970). A Manual for the Practical Study of the Root-Nodule Bacteria.

[47] Koeuth T., Versalovic J., Lupski J.R. (1995). Differential Subsequence Conservation of Interspersed Repetitive *Streptococcus pneumoniae* BOX Elements in Diverse Bacteria. Genome Res..

[48] Rivas R., Velázquez E., Valverde A., Mateos P.F., Martínez-Molina E. (2001). A Two Primers Random Amplified Polymorphic DNA Procedure to Obtain Polymerase Chain Reaction Fingerprints of Bacterial Species. Electrophoresis.

[49] Beckers B., Op De Beeck M., Thijs S., Truyens S., Weyens N., Boerjan W., Vangronsveld J. (2016). Performance of 16s RDNA Primer Pairs in the Study of Rhizosphere and Endosphere Bacterial Microbiomes in Metabarcoding Studies. Front. Microbiol..

[50] Dojka M.A., Hugenholtz P., Haack S.K., Pace N.R. (1998). Microbial Diversity in a Hydrocarbon- and Chlorinated-Solvent- Contaminated Aquifer Undergoing Intrinsic Bioremediation. Appl. Environ. Microbiol..

[51] Walker J.J., Pace N.R. (2007). Phylogenetic Composition of Rocky Mountain Endolithic Microbial Ecosystems. Appl. Environ. Microbiol..

[52] Altschul S.F., Gish W., Miller W., Myers E.W., Lipman D.J. (1990). Basic Local Alignment Search Tool. J. Mol. Biol..

[53] Chalita M., Kim Y.O., Park S., Oh H.-S., Cho J.H., Moon J., Baek N., Moon C., Lee K., Yang J. (2024). EzBioCloud: A Genome-Driven Database and Platform for Microbiome Identification and Discovery. Int. J. Syst. Evol. Microbiol..

[54] Hussain M.B., Zahir Z.A., Asghar H.N., Asgher M. (2014). Can Catalase and Exopolysaccharides Producing Rhizobia Ameliorate Drought Stress in Wheat?. Int. J. Agric. Biol..

[55] Johnson A.S., O’Sullivan E., D’Aoust L.N., Omer A., Bonner-Weir S., Fisher R.J., Weir G.C., Colton C.K. (2011). Quantitative Assessment of Islets of Langerhans Encapsulated in Alginate. Tissue Eng. Part C Methods.

[56] Gordon S.A., Weber R.P. (1951). Colorimetric Estimation of Indoleacetic Acid. Plant Physiol..

[57] Cardoso P., Alves A., Silveira P., Sá C., Fidalgo C., Freitas R., Figueira E. (2018). Bacteria from Nodules of Wild Legume Species: Phylogenetic Diversity, Plant Growth Promotion Abilities and Osmotolerance. Sci. Total Environ..

[58] Arora N.K., Verma M. (2017). Modified Microplate Method for Rapid and Efficient Estimation of Siderophore Produced by Bacteria. 3 Biotech.

[59] Nautiyal C.S. (1999). An Efficient Microbiological Growth Medium for Screening Phosphate Solubilizing Microorganisms. FEMS Microbiol. Lett..

[60] Pikovskaya R.I. (1948). Mobilization of Phosphorus in Soil Connection with the Vital Activity of Some Microbial Species. Microbiology.

[61] Murphy J., Riley J.P. (1962). A Modified Single Solution Method for the Determination of Phosphate in Natural Waters. Anal. Chim. Acta.

[62] Anderson M.J., Adam P. (2017). Permutational Multivariate Analysis of Variance (PERMANOVA). Wiley StatsRef: Statistics Reference Online.

[63] Anderson M.J., Walsh D.C.I. (2013). PERMANOVA, ANOSIM, and the Mantel Test in the Face of Heterogeneous Dispersions: What Null Hypothesis Are You Testing?. Ecol. Monogr..

[64] Chiarini L., Bevivino A., Dalmastri C., Nacamulli C., Tabacchioni S. (1998). Influence of Plant Development, Cultivar and Soil Type on Microbial Colonization of Maize Roots. Appl. Soil Ecol..

[65] Upadhyay S.K., Srivastava A.K., Rajput V.D., Chauhan P.K., Bhojiya A.A., Jain D., Chaubey G., Dwivedi P., Sharma B., Minkina T. (2022). Root Exudates: Mechanistic Insight of Plant Growth Promoting Rhizobacteria for Sustainable Crop Production. Front. Microbiol..

[66] Nyamwange M.M., Njeru E.M., Mucheru-Muna M. (2021). Tillage, Mulching and Nitrogen Fertilization Differentially Affects Soil Microbial Biomass, Microbial Populations and Bacterial Diversity in a Maize Cropping System. Front. Sustain. Food Syst..

[67] Houlden A., Timms-wilson T.M., Day M.J., Bailey M.J. (2008). Infuence of Plant Developmental Stage on Microbial Community Structure and Activity in the Rhizosphere of Three Field Crops. Fed. Eur. Microbiol. Soc..

[68] Hussain M.B., Mahmood S., Ahmed N., Nawaz H. (2018). Rhizobial Inoculation for Improving Growth Physiology, Nutrition and Yield of Maize under Drought Stress Conditions. Pak. J. Bot..

[69] Li Y., Li Q., Chen S. (2021). Diazotroph Paenibacillus Triticisoli BJ-18 Drives the Variation in Bacterial, Diazotrophic and Fungal Communities in the Rhizosphere and Root/Shoot Endosphere of Maize. Int. J. Mol. Sci..

[70] Niu B., Paulson J.N., Zheng X., Kolter R. (2017). Simplified and Representative Bacterial Community of Maize Roots. Proc. Natl. Acad. Sci. USA.

[71] Bai L., Wang Y., Li Y., Zhang X., Lu Z., Zhang D., Sun F., Zhao X. (2022). Changes in the Microbial Community in Maize (*Zea mays* L.) Root Spatial Structure Following Short-Term Nitrogen Application. ACS Omega.

[72] Chen L., Liu Y. (2024). The Function of Root Exudates in the Root Colonization by Beneficial Soil Rhizobacteria. Biology.

[73] Ofek M., Voronov-Goldman M., Hadar Y., Minz D. (2014). Host Signature Effect on Plant Root-associated Microbiomes Revealed through Analyses of Resident vs Active Communities. Environ. Microbiol..

[74] Beirinckx S., Viaene T., Haegeman A., Debode J., Amery F., Vandenabeele S., Nelissen H., Inzé D., Tito R., Raes J. (2020). Tapping into the Maize Root Microbiome to Identify Bacteria That Promote Growth under Chilling Conditions. Microbiome.

[75] Tiziani R., Miras-Moreno B., Malacrinò A., Vescio R., Lucini L., Mimmo T., Cesco S., Sorgonà A. (2022). Drought, Heat, and Their Combination Impact the Root Exudation Patterns and Rhizosphere Microbiome in Maize Roots. Environ. Exp. Bot..

[76] Mougel C., Offre P., Ranjard L., Corberand T., Gamalero E., Robin C., Lemanceau P. (2006). Dynamic of the Genetic Structure of Bacterial and Fungal Communities at Different Developmental Stages of *Medicago truncatula* Gaertn. cv. Jemalong line J5. New Phytol..

[77] Johnston-Monje D., Lundberg D.S., Lazarovits G., Reis V.M., Raizada M.N. (2016). Bacterial Populations in Juvenile Maize Rhizospheres Originate from Both Seed and Soil. Plant Soil.

[78] Lalande R., Bissonnette N., Coutlée D., Antoun H. (1989). Identification of Rhizobacteria from Maize and Determination of Their Plant-Growth Promoting Potential. Plant Soil.

[79] Wang P., Marsh E.L., Kruger G., Lorenz A., Schachtman D.P. (2020). Belowground Microbial Communities Respond to Water Deficit and Are Shaped by Decades of Maize Hybrid Breeding. Environ. Microbiol..

[80] Anzuay M.S., Viso N.P., Ludueña L.M., Morla F.D., Angelini J.G., Taurian T. (2021). Plant Beneficial Rhizobacteria Community Structure Changes through Developmental Stages of Peanut and Maize. Rhizosphere.

[81] Li J., Wang B., Ma H., Yang X., Wang T., Zhou G. (2025). Root Exudates Drive Plant-Microbiome Interactions Influencing the Quality of Cultivated *Rheum tanguticum* during Different Growth Development Stages. Ind. Crops Prod..

[82] Birt H.W.G., Tharp C.L., Custer G.F., Dini-andreote F. (2022). Root Phenotypes as Modulators of Microbial Microhabitats. Front. Plant Sci..

[83] Dadi F.A., Muthusamy S., Ghosh S., Muleta D., Tesfaye K., Assefa F. (2025). Plant Development Influences Dynamic Shifts in the Root Compartment Microbiomes of Wild and Domesticated Finger Millet Cultivars. BMC Microbiol..

[84] Rebollar E.A., Sandoval-Castellanos E., Roessler K., Gaut B.S., Alcaraz L.D., Benítez M., Escalante A.E. (2017). Seasonal Changes in a Maize-Based Polyculture of Central Mexico Reshape the Co-Occurrence Networks of Soil Bacterial Communities. Front. Microbiol..

[85] Afzal I., Shinwari Z.K., Sikandar S., Shahzad S. (2019). Plant Beneficial Endophytic Bacteria: Mechanisms, Diversity, Host Range and Genetic Determinants. Microbiol. Res..

[86] Molefe R.R., Amoo A.E., Babalola O.O. (2023). Communication between Plant Roots and the Soil Microbiome; Involvement in Plant Growth and Development. Symbiosis.

[87] Chaparro J.M., Badri D.V., Vivanco J.M. (2014). Rhizosphere Microbiome Assemblage Is Affected by Plant Development. ISME J..

[88] Liu Y., Xu C., Chen S., Chu G., Yu K., Wang D. (2025). Beyond Genotype: The Influence of Developmental Stage on Rice Rhizospheric Microbiome-Metabolome Networks. BMC Plant Biol..

[89] Santangeli M., Steininger-mairinger T., Vetterlein D., Hann S., Oburger E. (2024). Plant Science Maize (*Zea mays* L.) Root Exudation Profiles Change in Quality and Quantity during Plant Development—A Field Study. Plant Sci..

[90] Hafner B.D., Pietz O., King W.L., Scharfetter J.B., Bauerle T.L. (2025). Plant Science Early Developmental Shifts in Root Exudation Profiles of Five *Zea mays*. Plant Sci..

[91] Beauregard P.B. (2015). Not Just Sweet Talkers: How Roots Stimulate Their Colonization by Bene Fi Cial Bacteria.

[92] Shanahan J.F., Nielsen D.C. (1987). Influence of Growth Retardants (Anti-Gibberellins) on Corn Vegetative Growth, Water Use, and Grain Yield under Different Levels of Water Stress 1. Agron. J..

[93] Wang M., Zhao S., Wang L., Chen S., Li S., Lei X., Sun X., Qin L. (2021). Salt Stress-Induced Changes in Microbial Community Structures and Metabolic Processes Result in Increased Soil Cadmium Availability. Sci. Total Environ..

[94] Riah-Anglet W., Trinsoutrot-Gattin I., Martin-Laurent F., Laroche-Ajzenberg E., Norini M.P., Latour X., Laval K. (2015). Soil Microbial Community Structure and Function Relationships: A Heat Stress Experiment. Appl. Soil Ecol..

[95] Karagulyan M., Goebel M.O., Diehl D., Quba A.A.A., Kästner M., Bachmann J., Wick L.Y., Schaumann G.E., Miltner A. (2022). Water Stress-Driven Changes in Bacterial Cell Surface Properties. Appl. Environ. Microbiol..

[96] Sharma I., Kashyap S., Agarwala N. (2023). Biotic Stress-Induced Changes in Root Exudation Confer Plant Stress Tolerance by Altering Rhizospheric Microbial Community. Front. Plant Sci..

[97] Rangarajan S., Saleena L.M., Nair S. (2002). Diversity of *Pseudomonas* Spp. Isolated from Rice Rhizosphere Populations Grown along a Salinity Gradient. Microb. Ecol..

[98] Nicotra D., Mosca A., Dimaria G., Massimino M.E., Di Stabile M., La Bella E., Ghadamgahi F., Puglisi I., Vetukuri R.R., Catara V. (2025). Mitigating Water Stress in Plants with Beneficial Bacteria: Effects on Growth and Rhizosphere Bacterial Communities. Int. J. Mol. Sci..

[99] Liu K., Deng F., Zeng F., Yuan Z.C., Guang Q. (2025). Plant Growth-Promoting Rhizobacteria Improve Drought Tolerance of Crops: A Review. Plant Growth Regul.

[100] Ahmed B., Shahid M., Syed A., Rajput V.D., Elgorban A.M., Minkina T., Bahkali A.H., Lee J. (2021). Drought Tolerant *Enterobacter* Sp.*/Leclercia adecarboxylata* Secretes Indole-3-Acetic Acid and Other Biomolecules and Enhances the Biological Attributes of *Vigna radiata* (L.) R. Wilczek in Water Deficit Conditions. Biology.

[101] Muñoz-carvajal E., Fuentes Y., Oetiker N., Giordano A., Stoll A. (2024). Rhizobacteria *Enterobacter* Sp. LHB11 and *Bacillus* Sp. PIXIE Induced Systemic Tolerance Against Drought Stress in Tomato (*Solanum lycopersicum*). Agronomy.

[102] Feng C., Li J., Qin D., Chen L., Zhao F., Chen S., Hu H., Yu C. (2014). Characterization of Exoelectrogenic Bacteria *Enterobacter* Strains Isolated from a Microbial Fuel Cell Exposed to Copper Shock Load. PLoS ONE.

[103] Vescio R., Malacrinò A., Bennett A.E., Sorgonà A. (2021). Single and Combined Abiotic Stressors Affect Maize Rhizosphere Bacterial Microbiota. Rhizosphere.

[104] Zhang Z., Wang D., Li M. (2022). Soil Respiration, Aggregate Stability and Nutrient Availability Affected by Drying Duration and Drying-Rewetting Frequency. Geoderma.

[105] Yan N., Marschner P., Cao W., Zuo C., Qin W. (2015). Influence of Salinity and Water Content on Soil Microorganisms. Int. Soil Water Conserv. Res..

[106] Abdelaal K., AlKahtani M., Attia K., Hafez Y., Király L., Künstler A. (2021). The Role of Plant Growth-Promoting Bacteria in Alleviating the Adverse Effects of Drought on Plants. Biology.

[107] Wichern J., Wichern F., Joergensen R.G. (2006). Impact of Salinity on Soil Microbial Communities and the Decomposition of Maize in Acidic Soils. Geoderma.

[108] Pereira L.B., de Oliveira Gambarini V.M., de Menezes A.B., Ottoboni L.M.M., Vicentini R. (2021). Responses of the Sugarcane Rhizosphere Microbiota to Different Levels of Water Stress. Appl. Soil Ecol..

[109] Rojas-Tapias D., Moreno-Galván A., Pardo-Díaz S., Obando M., Rivera D., Bonilla R. (2012). Effect of Inoculation with Plant Growth-Promoting Bacteria (PGPB) on Amelioration of Saline Stress in Maize (*Zea mays*). Appl. Soil Ecol..

[110] Bandeppa G.S., Sangeeta P., Chetana A., Manjunatha B.S., Rathi M.S. (2018). Characterization of Osmotolerant Rhizobacteria for Plant Growth Promoting Activities in Vitro and during Plant-Microbe Association under Osmotic Stress. Indian J. Exp. Biol..

[111] Sandhya V., Ali S.Z., Venkateswarlu B., Reddy G., Grover M. (2010). Effect of Osmotic Stress on Plant Growth Promoting *Pseudomonas* Spp.. Arch. Microbiol..

[112] Devarajan A.K., Truu M., Gopalasubramaniam S.K., Muthukrishanan G., Truu J. (2022). Application of Data Integration for Rice Bacterial Strain Selection by Combining Their Osmotic Stress Response and Plant Growth-Promoting Traits. Front. Microbiol..

[113] Ikeda A.C., Savi D.C., Hungria M., Kava V., Glienke C., Galli-Terasawa L.V. (2020). Bioprospecting of Elite Plant Growth-Promoting Bacteria for the Maize Crop. Acta Sci.—Agron..

[114] Sá C., Cardoso P., Figueira E. (2019). Alginate as a Feature of Osmotolerance Differentiation among Soil Bacteria Isolated from Wild Legumes Growing in Portugal. Sci. Total Environ..

[115] Sandhya V., Ali S.Z., Grover M., Reddy G., Venkateswarlu B. (2009). Alleviation of Drought Stress Effects in Sunflower Seedlings by the Exopolysaccharides Producing *Pseudomonas putida* Strain GAP-P45. Biol. Fertil. Soils.

[116] Chang W.S., Van De Mortel M., Nielsen L., De Guzman G.N., Li X., Halverson L.J. (2007). Alginate Production by *Pseudomonas putida* Creates a Hydrated Microenvironment and Contributes to Biofilm Architecture and Stress Tolerance under Water-Limiting Conditions. J. Bacteriol..

[117] Liu H., Zhang Y.-H., Yin H., Wang W.-X., Zhao X.-M., Du Y.-G. (2013). Alginate Oligosaccharides Enhanced *Triticum aestivum* L. Tolerance to Drought Stress. Plant Physiol. Biochem..

[118] Li Z., Zhang X., Zhao Y., Li Y., Zhang G., Peng Z., Zhang J. (2018). Enhancing Auxin Accumulation in Maize Root Tips Improves Root Growth and Dwarfs Plant Height. Plant Biotechnol. J..

[119] Etesami H., Glick B.R. (2024). Bacterial Indole-3-Acetic Acid: A Key Regulator for Plant Growth, Plant-Microbe Interactions, and Agricultural Adaptive Resilience. Microbiol. Res..

[120] Sadeghi A., Karimi E., Dahaji P.A., Javid M.G., Dalvand Y., Askari H. (2012). Plant Growth Promoting Activity of an Auxin and Siderophore Producing Isolate of *Streptomyces* under Saline Soil Conditions. World J. Microbiol. Biotechnol..

[121] Singh P., Chauhan P.K., Upadhyay S.K., Singh R.K., Dwivedi P., Wang J., Jain D., Jiang M. (2022). Mechanistic Insights and Potential Use of Siderophores Producing Microbes in Rhizosphere for Mitigation of Stress in Plants Grown in Degraded Land. Front. Microbiol..

[122] Rai S., Singh P.K., Mankotia S., Swain J., Satbhai S.B. (2021). Iron Homeostasis in Plants and Its Crosstalk with Copper, Zinc, and Manganese. Plant Stress.

[123] Siddique S., Naveed M., Yaseen M., Shahbaz M. (2022). Exploring Potential of Seed Endophytic Bacteria for Enhancing Drought Stress Resilience in Maize (*Zea mays* L.). Sustainability.

[124] Kour D., Rana K.L., Kaur T., Sheikh I., Yadav A.N., Kumar V., Dhaliwal H.S., Saxena A.K. (2020). Microbe-Mediated Alleviation of Drought Stress and Acquisition of Phosphorus in Great Millet (*Sorghum bicolour* L.) by Drought-Adaptive and Phosphorus-Solubilizing Microbes. Biocatal. Agric. Biotechnol..

[125] Vardharajula S., Zulfikar Ali S., Grover M., Reddy G., Bandi V. (2011). Drought-Tolerant Plant Growth Promoting *Bacillus* Spp.: Effect on Growth, Osmolytes, and Antioxidant Status of Maize under Drought Stress. J. Plant Interact..

[126] Kour D., Rana K.L., Sheikh I., Kumar V., Yadav A.N., Dhaliwal H.S., Saxena A.K. (2020). Alleviation of Drought Stress and Plant Growth Promotion by *Pseudomonas libanensis* EU-LWNA-33, a Drought-Adaptive Phosphorus-Solubilizing Bacterium. Proc. Natl. Acad. Sci. India Sect. B Biol. Sci..

[127] Kour D., Rana K.L., Yadav A.N., Sheikh I., Kumar V., Dhaliwal H.S., Saxena A.K. (2020). Amelioration of Drought Stress in Foxtail Millet (*Setaria italica* L.) by P-Solubilizing Drought-Tolerant Microbes with Multifarious Plant Growth Promoting Attributes. Environ. Sustain..

[128] Adnan M., Fahad S., Zamin M., Shah S., Mian I.A., Danish S., Zafar-ul-Hye M., Battaglia M.L., Naz R.M.M., Saeed B. (2020). Coupling Phosphate-Solubilizing Bacteria with Phosphorus Supplements Improve Maize Phosphorus Acquisition and Growth under Lime Induced Salinity Stress. Plants.

[129] Egamberdiyeva D. (2007). The Effect of Plant Growth Promoting Bacteria on Growth and Nutrient Uptake of Maize in Two Different Soils. Appl. Soil Ecol..

[130] Olanrewaju O.S., Babalola O.O. (2019). Bacterial Consortium for Improved Maize (*Zea mays* L.) Production. Microorganisms.

