# Supplementary material for: Influence of Plant Developmental Phase and Irrigation Level on Cultivable Microbiome of Maize Root
Source: Biology (Basel). 2025 Nov 28;14(12):1694. doi: 10.3390/biology14121694 (PMC12730274; doi:10.3390/biology14121694)
Supplement: Supplementary file 1 [file biology-14-01694-s001.zip › biology-3974875-supplementary.pdf]

Table S1. Statistical analysis of endophytic bacterial plant growth-promoting traits under control (0% of polyethylene glycol (PEG) 6000) and osmotic stress (15% of PEG 6000) conditions. Growth phases are denoted as V (vegetative) and R (reproductive), and irrigation levels applied to the sampled roots are indicated as 100%, 50%, and 0%. The table includes the test statistic (t), permutational p-values (P(perm)), number of unique permutations (Unique perms), and Monte Carlo p-values (P(MC)), where applicable.

Endophytic bacteria	Alginate				Indole-3-acetic acid				Siderophores				Phosphate			
Groups	t	P(perm)	Unique perms	P(MC)	t	P(perm)	Unique perms	P(MC)	t	P(perm)	Unique perms	P(MC)	t	P(perm)	Unique perms	P(MC)
V_0%PEG_100%, V_0%PEG_50%	1,4138	0,1643	9833	0,1656	0,3062	0,9604	9955	0,7621	0,19364	0,8514	8349	0,8512	0,24026	0,8064	9794	0,8014
V_0%PEG_100%, V_0%PEG_0%	1,5056	0,1454	9846	0,1436	0,78298	0,5778	9932	0,4407	1,5051	0,1388	9042	0,1502	0,71903	0,4646	9834	0,4838
V_0%PEG_50%, V_0%PEG_0%	0,24923	0,8109	9849	0,8038	2,3475	0,0301	9819	0,0264	1,4023	0,1675	8920	0,1747	0,85347	0,3969	9846	0,4003
V_15%PEG_100%, V_15%PEG_50%	2,2146	0,0346	9835	0,0317	0,36404	0,7062	9840	0,7112	0,46229	0,6549	8950	0,6439	0,49252	0,6244	9816	0,6213
V_15%PEG_100%, V_15%PEG_0%	1,8892	0,0622	9839	0,0716	0,39234	0,6819	9823	0,6941	0,49846	0,6162	9252	0,6213	0,16461	0,8681	9767	0,8679
V_15%PEG_50%, V_15%PEG_0%	7,2074E-2	0,9452	9839	0,9446	1,4321	0,163	9842	0,1601	0,81321	0,4249	9342	0,415	0,3143	0,7649	9700	0,7495
V_0%PEG_100%, V_15%PEG_100%	1,7988	0,0755	9863	0,0751	0,3856	0,7628	9892	0,7027	6,7678	0,0001	9809	0,0001	0,92806	0,3573	9829	0,3595
V_0%PEG_50%, V_15%PEG_50%	1,9725	0,0543	9831	0,0612	0,92466	0,3603	9834	0,3649	6,3529	0,0001	9845	0,0001	0,59066	0,555	9834	0,5507
V_0%PEG_0%, V_15%PEG_0%	1,6537	0,1038	9881	0,1121	3,5439E-2	0,9713	9837	0,9714	6,1438	0,0002	9843	0,0001	Negative			
V_0%PEG_100%, R_0%PEG_100%	0,16877	0,8721	9841	0,8631	0,31802	0,83	9911	0,7531	1,8824	0,0673	9135	0,0656	2,8764	0,0066	9802	0,0041
V_0%PEG_50%, R_0%PEG_50%	0,42314	0,6852	9849	0,6843	0,91449	0,3645	9850	0,3698	1,0841	0,2812	9000	0,2881	1,2734	0,213	9739	0,2061
V_0%PEG_0%, R_0%PEG_0%	1,3526	0,22	9800	0,1821	8,275E-2	0,9312	9828	0,9326	0,32684	0,7382	9064	0,7401	0,34091	0,74	9824	0,7388
R_0%PEG_100%, R_0%PEG_50%	2,1485	0,0353	9829	0,037	0,63805	0,5271	9835	0,5237	0,69189	0,4999	9454	0,4915	4,3965	0,0001	9768	0,0002
R_0%PEG_100%, R_0%PEG_0%	2,5856	0,0114	9865	0,0141	0,89226	0,3773	9829	0,372	0,4905	0,6353	9036	0,6349	1,2887	0,1982	9774	0,2001
R_0%PEG_50%, R_0%PEG_0%	1,1148	0,2791	9855	0,2715	1,5399	0,136	9827	0,1356	9,1808E-2	0,9284	9021	0,9265	2,0499	0,0446	9794	0,0465
R_15%PEG_100%, R_15%PEG_50%	0,51633	0,6328	9856	0,6058	1,6737	0,1016	9842	0,1022	3,3909	0,0012	9614	0,0011	0,99251	0,319	9747	0,3307
R_15%PEG_100%, R_15%PEG_0%	0,19333	0,8532	9842	0,8429	2,5494	0,0137	9859	0,0138	1,485	0,1405	9685	0,1395	0,64031	0,5272	9629	0,5233
R_15%PEG_50%, R_15%PEG_0%	0,29962	0,7741	9853	0,7726	3,8334	0,0003	9855	0,0006	1,6882	0,1014	9715	0,0977	1,6525	0,1044	9720	0,1046
R_0%PEG_100%, R_15%PEG_100%	2,8492	0,0004	9876	0,0055	1,6389	0,1068	9822	0,1051	4,0129	0,0005	9849	0,0001	1,2193	0,2165	9836	0,2259
R_0%PEG_50%, R_15%PEG_50%	2,5342	0,0055	9874	0,0148	0,83626	0,4066	9839	0,4018	8,3803	0,0001	9834	0,0001	3,2372	0,0021	9847	0,0021
R_0%PEG_0%, R_15%PEG_0%	1,3413	0,1926	9866	0,1908	2,1393	0,0445	9835	0,0389	4,6838	0,0001	9837	0,0001	0,90341	0,3624	9831	0,3689
V_15%PEG_100%, R_15%PEG_100%	2,3502	0,0082	9860	0,0232	0,70161	0,4874	9832	0,4959	3,6705	0,0005	9665	0,0004	1,1158	0,264	9830	0,2729
V_15%PEG_50%, R_15%PEG_50%	0,79681	0,4605	9850	0,4374	0,37843	0,6985	9829	0,7032	0,72403	0,4717	9489	0,471	0,91994	0,3572	9617	0,3611
V_15%PEG_0%, R_15%PEG_0%	0,9646	0,348	9858	0,3468	2,4273	0,0199	9851	0,0248	1,2406	0,22	9659	0,2255	1,7197	0,0914	9533	0,0939

Table S2. Statistical analysis of the rhizoplane bacterial plant growth-promoting (PGP) traits under control (0% of polyethylene glycol (PEG) 6000) and osmotic stress (15% of PEG 6000) conditions. Growth phases are denoted as V (vegetative) and R (reproductive), and irrigation levels applied to the sampled roots are indicated as 100%, 50%, and 0%. The table includes the test statistic (t), permutational p-values (P(perm)), number of unique permutations (Unique perms), and Monte Carlo p-values (P(MC)), where applicable.

Rhizoplane bacteria	Alginate				Indole acetic acid				Siderophores				Phosphate			
Groups	t	P(perm)	Unique perms	P(MC)	t	P(perm)	Unique perms	P(MC)	t	P(perm)	Unique perms	P(MC)	t	P(perm)	Unique perms	P(MC)
V_0%PEG_100%, V_0%PEG_50%	0,76328	0,5454	9909	0,4509	0,45734	0,6555	9831	0,6438	1,8014	0,0714	9785	0,0821	0,40201	0,6903	9634	0,6973
V_0%PEG_100%, V_0%PEG_0%	0,38691	0,7607	9882	0,7004	1,7982	0,0721	9837	0,077	2,3479	0,0215	9571	0,0218	1,2451	0,2105	9669	0,2197
V_0%PEG_50%, V_0%PEG_0%	0,47821	0,6515	9857	0,6302	1,5704	0,1232	9844	0,1204	0,81453	0,4249	9392	0,425	1,6585	0,1022	9571	0,1012
V_15%PEG_100%, V_15%PEG_50%	0,30314	0,771	9824	0,7593	5,2433E-2	0,9579	9825	0,9567	3,4012	0,0014	9773	0,0015	2,3823E-2	0,9812	9582	0,9814
V_15%PEG_100%, V_15%PEG_0%	2,8607	0,0017	9850	0,0049	0,68904	0,6086	9906	0,4896	3,8141	0,0005	9309	0,0004	1,0835	0,2834	9476	0,2796
V_15%PEG_50%, V_15%PEG_0%	3,3358	0,0002	9851	0,0015	0,67162	0,6015	9940	0,5083	0,10005	0,9273	9592	0,9238	1,0575	0,2903	9566	0,2974
V_0%PEG_100%, V_15%PEG_100%	1,5695	0,1261	9880	0,1241	1,5247	0,1365	9821	0,1272	6,7959	0,0001	9833	0,0001	0,20215	0,8388	9820	0,8404
V_0%PEG_50%, V_15%PEG_50%	2,4372	0,008	9859	0,0186	1,1896	0,2441	9844	0,2366	6,0577	0,0001	9846	0,0001	0,31145	0,7588	9840	0,7557
V_0%PEG_0%, V_15%PEG_0%	3,1891	0,0001	9865	0,0019	3,5494E-3	0,9977	9883	0,9973	7,0545	0,0001	9858	0,0001	2,3596	0,0207	9818	0,0225
V_0%PEG_100%, R_0%PEG_100%	1,4136	0,1682	9849	0,1674	1,4022	0,164	9831	0,164	1,9198	0,0551	9557	0,055	2,2836	0,0264	9848	0,0238
V_0%PEG_50%, R_0%PEG_50%	2,4745	0,0129	9826	0,0158	0,9573	0,3489	9848	0,3459	2,9119E-2	0,9788	9786	0,9781	2,7201	0,0071	9824	0,0065
V_0%PEG_0%, R_0%PEG_0%	1,7744	0,0099	9955	0,0805	0,70885	0,518	9855	0,4783	1,0968	0,2819	9745	0,2756	1,1476	0,2509	9661	0,2599
R_0%PEG_100%, R_0%PEG_50%	0,8196	0,422	9833	0,4086	0,11518	0,9091	9835	0,9106	9,7172E-2	0,9254	9702	0,924	0,26858	0,7849	9822	0,7947
R_0%PEG_100%, R_0%PEG_0%	1,5123	0,1058	9912	0,1361	1,9796	0,0286	9871	0,0506	0,68716	0,5016	9724	0,4957	2,1898	0,0304	9765	0,0327
R_0%PEG_50%, R_0%PEG_0%	1,806	0,0236	9952	0,077	1,8328	0,0581	9893	0,0717	0,49913	0,6213	9760	0,6222	2,2486	0,0257	9833	0,0308
R_15%PEG_100%, R_15%PEG_50%	0,89357	0,7288	9900	0,3772	0,59598	0,5634	9836	0,5475	0,76692	0,4537	9794	0,4488	3,934	0,0003	9771	0,0002
R_15%PEG_100%, R_15%PEG_0%	1,0363	0,027	9313	0,296	2,8526	0,0052	9824	0,0047	0,20721	0,8357	9779	0,8396	0,95608	0,333	9663	0,3435
R_15%PEG_50%, R_15%PEG_0%	0,96481	0,5051	9684	0,3331	2,1818	0,0337	9824	0,032	0,55255	0,5887	9834	0,5799	2,8453	0,0067	9725	0,005
R_0%PEG_100%, R_15%PEG_100%	4,1407	0,0001	9853	0,0001	0,46782	0,6494	9863	0,6347	6,8705	0,0001	9840	0,0001	1,5127	0,1368	9844	0,1331
R_0%PEG_50%, R_15%PEG_50%	1,3831	0,0002	9878	0,1722	0,85436	0,398	9830	0,3956	4,1742	0,0003	9852	0,0001	1,0418	0,2992	9835	0,3
R_0%PEG_0%, R_15%PEG_0%	0,87109	0,4175	9651	0,383	0,58415	0,5849	9866	0,5593	4,2179	0,0001	9827	0,0002	1,8567	0,0706	9853	0,0709
V_15%PEG_100%, R_15%PEG_100%	3,0839	0,0019	9831	0,0037	0,24392	0,8166	9853	0,8097	2,1594	0,0303	9757	0,034	1,9836	0,0499	9745	0,0525
V_15%PEG_50%, R_15%PEG_50%	1,242	0,04	9903	0,2194	0,7525	0,4571	9839	0,4648	2,947E-2	0,9785	9831	0,9768	6,0095	0,0001	9699	0,0001
V_15%PEG_0%, R_15%PEG_0%	0,79497	0,7631	9657	0,4303	1,2212	0,2322	9834	0,224	0,76399	0,4528	9688	0,452	3,4285	0,001	9605	0,0013

Table S3. Statistical analysis of the colony-forming units (CFU) data, based on the averages of technical replicates (Table S4). Growth phases are denoted as V (vegetative) and R (reproductive), and the irrigation levels applied to the sampled soil are indicated as 100%, 50%, and 0%. The table includes the test statistic (t), permutational p-values (P(perm)), number of unique permutations (Unique perms), and Monte Carlo p-values (P(MC)), where applicable.

	Statistical analysis with the average of the technical replicate			
Groups	t	P(perm)	Unique perms	P(MC)
V_100%, V_50%	1,1456	0,3202	126	0,2881
V_100%, V_0%	0,59922	0,5689	126	0,5622
V_50%, V_0%	0,60875	0,6101	126	0,5576
R_100%, R_50%	0,82876	0,429	126	0,4338
R_100%, R_0%	0,30847	0,7741	126	0,7603
R_50%, R_0%	0,6385	0,5339	126	0,5455
V_100%, R_100%	4,8797	0,0101	126	0,0016
V_50%, R_50%	3,7332	0,017	126	0,0048
V_0%, R_0%	5,2242	0,0091	126	0,0012

Table S4. Raw colony-forming unit (CFU) data. The letter r denotes biological replicates, and irrigation levels applied to the sampled roots are indicated as 100%, 50%, and 0%. The suffixes _1 to _5 represent the technical replicates of each biological sample.

10^5 CFU/g of soil	1º sample (Vegetative phase)			2º sample (Reproductive phase)			1º sample	2º sample
sample	r1	r2	r3	r1	r2	r3	average	average
100%_1	62.73651	70.70305	56.7616	162.3183	128.4605	126.4688	63.40	139.08
100%_2	38.25341	46.65049	101.6981	107.2961	95.16701	91.43497	62.20	97.97
100%_3	21.84056	37.72461	42.68837	122.1086	139.9782	131.0434	34.08	131.04
100%_4	17.9737	37.83937	19.86567	157.0334	108.7882	117.3021	25.23	127.71
100%_5	41.58416	49.50495	52.47525	199.0099	223.7624	192.0792	47.85	204.95
50%_1	43.30282	50.19191	41.33451	228.324	217.4983	209.625	44.94	218.48
50%_2	205.6606	158.6524	24.4834	106.7476	115.5616	103.8096	129.60	108.71
50%_3	71.89993	73.86979	55.15611	214.7149	175.3176	160.5437	66.98	183.53
50%_4	42.32283	51.1811	20.66929	304.1339		31.49606	38.06	167.81
50%_5	106.5313	22.89924	39.82477	126.4436	123.4568	142.3736	56.42	130.76
0%_1	48.39028	61.22852	30.61426	146.1584	211.3372	177.7602	46.74	178.42
0%_2	69.02672	27.61069	43.38823	112.4149	176.5112	154.8171	46.68	147.91
0%_3	123.9752	49.99	82.9834	137.9724	139.972	240.9518	85.65	172.97
0%_4	29.92519	16.95761	11.97007	143.6409	101.7456	122.6933	19.62	122.69
0%_5	74.65619	74.65619	78.58546	119.8428	92.33792	125.7367	75.97	112.64

Table S5. GenBank accession numbers and identification at the genus/family level. Most isolates were amplified using the 799F and 1492R primers, except for isolates PP150900, OR948112, OR948113, OR948114, OR948115, PP150899, and OR948111, which were amplified with the 799F and 1391R.

Accession number	Identification
**OR948386**	*Limnohabitans* sp.
**OR948376**	*Limnohabitans* sp.
**OR948158**	*Pseudomonas* sp.
**OR948157**	*Pseudomonas* sp.
**OR948159**	*Pseudomonas* sp.
**OR948267**	*Pseudomonas* sp.
**OR948234**	*Delftia* sp.
**PP150906**	Rhizobiaceae
**OR948246**	*Priestia* sp.
**OR948214**	*Delftia* sp.
**OR948278**	*Herbaspirillum* sp.
**OR948268**	*Herbaspirillum* sp.
**OR948338**	*Pseudomonas* sp.
**OR948327**	*Massilia* sp.
**OR948400**	*Pantoea* sp.
**OR948235**	*Delftia* sp.
**OR948225**	*Delftia* sp.
**OR948348**	*Sphingomonas* sp.
**OR948391**	*Stenotrophomonas* sp.
**OR948287**	*Pseudomonas* sp.
**OR948279**	*Pseudomonas* sp.
**OR948215**	*Pseudomonas* sp.
**OR948369**	*Stenotrophomonas* sp.
**OR948233**	*Pseudomonas* sp.
**OR948224**	*Pseudomonas* sp.
**OR948213**	*Bosea* sp.
**OR948097**	*Paenarthrobacter* sp.
**OR948098**	*Acidovorax* sp.
**OR948314**	*Pseudomonas* sp.
**OR948207**	*Flavobacterium* sp.
**OR948273**	*Burkholderia* sp.
**OR948261**	*Pseudomonas* sp.
**OR948250**	*Achromobacter* sp.
**OR948240**	*Burkholderia* sp.
**OR948315**	*Pseudomonas* sp.
**OR948316**	*Pseudomonas* sp.
**OR948228**	*Pseudomonas* sp.
**OR948229**	*Flavobacterium* sp.

**OR948219**	*Pseudomonas* sp.
**OR948218**	*Pseudomonas* sp.
**OR948183**	*Flavobacterium* sp.
**OR948396**	*Pseudomonas* sp.
**PP150903**	Rhizobiaceae
**OR948217**	*Cedecea* sp.
**OR948319**	*Stenotrophomonas* sp.
**OR948206**	*Pseudomonas* sp.
**OR948260**	*Agrobacterium* sp.
**OR948249**	*Agrobacterium* sp.
**OR948239**	*Pseudomonas* sp.
**OR948189**	*Pseudomonas* sp.
**PP150908**	Rhizobiaceae
**OR948272**	*Pseudomonas*
**OR948190**	*Enterobacter* sp.
**OR948192**	*Pseudomonas* sp.
**OR948193**	*Enterobacter* sp.
**OR948194**	*Enterobacter* sp.
**PP150902**	Enterobacteriaceae
**OR948195**	*Enterobacter* sp.
**OR948196**	Lysobacteraceae
**OR948197**	*Pseudomonas* sp.
**OR948198**	*Cedecea* sp.
**OR948191**	*Pseudomonas* sp.
**OR948201**	*Pseudomonas* sp.
**OR948202**	*Pseudomonas* sp.
**OR948162**	*Pseudomonas* sp.
**OR948163**	*Microbacterium* sp.
**OR948199**	*Pseudomonas* sp.
**OR948200**	*Pseudomonas* sp.
**OR948174**	*Flavobacterium* sp.
**OR948166**	*Pseudomonas* sp.
**OR948164**	*Pseudomonas* sp.
**OR948167**	*Enterobacter* sp.
**OR948184**	*Cedecea* sp.
**OR948165**	*Microbacterium* sp.
**OR948186**	*Stenotrophomonas* sp.
**OR948187**	*Stenotrophomonas* sp.
**OR948188**	*Stenotrophomonas* sp.
**OR948185**	*Stenotrophomonas* sp.
**OR948220**	*Scandinavium* sp.
**OR948209**	*Curtobacterium* sp.
**OR948283**	*Flavobacterium* sp.

**OR948275**	*Acinetobacter* sp.
**OR948263**	*Pseudomonas* sp.
**OR948274**	*Flavobacterium* sp.
**OR948262**	*Stenotrophomonas* sp.
**OR948282**	*Pseudomonas* sp.
**OR948230**	*Pseudomonas* sp.
**OR948252**	*Pseudomonas* sp.
**OR948104**	*Enterobacter* sp.
**OR948105**	*Enterobacter* sp.
**OR948106**	*Enterobacter* sp.
**OR948107**	*Enterobacter* sp.
**OR948108**	*Enterobacter* sp.
**PP150904**	Brucellaceae
**OR948109**	*Enterobacter* sp.
**OR948384**	*Pseudomonas* sp.
**OR948117**	*Pseudomonas* sp.
**OR948382**	*Enterobacter* sp.
**OR948374**	*Pseudomonas* sp.
**OR948110**	*Enterobacter* sp.
**OR948118**	*Enterobacter* sp.
**OR948394**	*Priestia* sp.
**PP150909**	Rhizobiaceae
**PP150916**	Enterobacteriaceae
**PP150913**	*Erwinia* sp.
**OR948182**	*Pseudomonas* sp.
**OR948181**	*Pseudomonas* sp.
**OR948161**	*Flavobacterium* sp.
**OR948172**	*Pseudomonas* sp.
**OR948155**	*Pseudomonas* sp.
**OR948154**	*Pseudomonas* sp.
**OR948231**	*Pseudomonas* sp.
**OR948276**	*Pseudomonas* sp.
**OR948264**	*Pseudomonas* sp.
**OR948253**	*Pseudomonas* sp.
**OR948221**	*Enterobacter* sp.
**OR948210**	*Pseudomonas* sp.
**OR948284**	*Pseudomonas* sp.
**OR948145**	*Enterobacter* sp.
**OR948149**	*Enterobacter* sp.
**OR948146**	*Enterobacter* sp.
**OR948147**	*Enterobacter* sp.
**OR948148**	*Enterobacter* sp.
**OR948344**	*Priestia* sp.

**OR948255**	*Priestia* sp.
**OR948244**	*Brucella* sp.
**OR948333**	*Neorhizobium* sp.
**OR948393**	*Bacillus* sp.
**PP150921**	Enterobacteriaceae
**OR948223**	*Priestia* sp.
**OR948212**	*Pseudomonas* sp.
**OR948241**	*Arthrobacter* sp.
**OR948266**	*Agrobacterium* sp.
**OR948257**	*Enterobacter* sp.
**OR948247**	*Enterobacter* sp.
**OR948288**	*Bradyrhizobium* sp.
**OR948236**	*Enterobacter* sp.
**OR948383**	*Pseudomonas* sp.
**OR948280**	*Pantoea* sp.
**OR948269**	*Priestia* sp.
**OR948281**	*Microbacterium* sp.
**OR948151**	*Pseudomonas* sp.
**OR948270**	*Brucella* sp.
**PP150912**	Rhizobiaceae
**OR948152**	*Pseudomonas* sp.
**OR948340**	*Chryseobacterium* sp.
**OR948226**	*Priestia* sp.
**OR948216**	*Mesorhizobium* sp.
**OR948204**	*Priestia* sp.
**OR948329**	*Enterobacter* sp.
**OR948245**	*Pseudoxanthomonas* sp.
**OR948403**	*Paraburkholderia* sp.
**OR948325**	*Pantoea* sp.
**OR948321**	*Pantoea* sp.
**OR948256**	*Priestia* sp.
**OR948173**	*Flavobacterium* sp.
**OR948134**	*Pseudomonas* sp.
**OR948208**	*Stenotrophomonas* sp.
**OR948133**	*Pseudomonas* sp.
**OR948128**	*Pseudomonas* sp.
**OR948120**	*Cedecea* sp.
**OR948121**	*Bacillus* sp.
**OR948122**	*Bacillus* sp.
**OR948130**	*Enterobacter* sp.
**OR948135**	*Pseudomonas* sp.
**OR948119**	*Curtobacterium* sp.
**OR948312**	*Flavobacterium* sp.

**PP150901**	Rhizobiaceae
**OR948123**	*Pseudomonas* sp.
**OR948124**	*Cedecea* sp.
**OR948125**	*Cedecea* sp.
**OR948126**	*Pseudomonas* sp.
**OR948099**	*Enterobacter* sp.
**OR948101**	*Enterobacter* sp.
**OR948102**	Enterobacter sp.
**OR948103**	*Cedecea* sp.
**OR948129**	*Enterobacter* sp.
**OR948100**	*Enterobacter* sp.
**OR948168**	*Enterobacter* sp.
**OR948170**	*Bacillus* sp.
**OR948127**	*Enterobacter* sp.
**OR948169**	*Enterobacter* sp.
**OR948132**	*Pantoea* sp.
**OR948131**	*Enterobacter* sp.
**OR948171**	*Pantoea* sp.
**OR948222**	*Stenotrophomonas* sp.
**OR948211**	*Stenotrophomonas* sp.
**OR948242**	*Bradyrhizobium* sp.
**OR948232**	*Pantoea* sp.
**OR948285**	*Pantoea* sp.
**OR948265**	*Bacillus* sp.
**OR948254**	*Agrobacterium* sp.
**OR948243**	*Agrobacterium* sp.
**OR948277**	*Bacillus* sp.
**OR948153**	*Pseudomonas* sp.
**OR948096**	*Priestia* sp.
**OR948313**	*Enterobacter* sp.
**OR948095**	*Priestia* sp.
**OR948258**	*Paraburkholderia* sp.
**OR948227**	*Agrobacterium* sp.
**OR948205**	*Variovorax* sp.
**OR948271**	*Enterobacter* sp.
**OR948259**	*Agrobacterium* sp.
**OR948248**	*Variovorax* sp.
**OR948238**	*Cedecea* sp.
**OR948076**	*Cedecea* sp.
**PP150905**	Rhizobiaceae
**OR948077**	*Enterobacter* sp.
**OR948078**	*Burkholderia* sp.
**OR948237**	*Pseudomonas* sp.

**OR948083**	*Enterobacter* sp.
**OR948084**	*Cedecea* sp.
**OR948085**	*Cedecea* sp.
**OR948069**	*Pseudomonas* sp.
**OR948070**	*Pseudomonas* sp.
**OR948079**	*Cedecea* sp.
**OR948071**	*Pseudomonas* sp.
**OR948072**	*Pantoea* sp.
**OR948080**	*Enterobacter* sp.
**OR948081**	*Cedecea* sp.
**OR948082**	*Cedecea* sp.
**OR948074**	*Pantoea* sp.
**OR948073**	*Pantoea* sp.
**OR948057**	*Enterobacter* sp.
**OR948060**	*Pseudomonas* sp.
**OR948061**	*Microbacterium* sp.
**PP150900**	*Pseudomonas* sp.
**OR948112**	*Pseudomonas* sp.
**OR948113**	*Pseudomonas* sp.
**OR948114**	*Pseudomonas* sp.
**OR948115**	*Bosea* sp.
**OR948251**	*Pseudomonas* sp.
**OR948364**	*Pseudomonas* sp.
**OR948370**	*Achromobacter* sp.
**OR948058**	*Cedecea* sp.
**OR948059**	*Pseudomonas* sp.
**OR948360**	*Arthrobacter* sp.
**OR948381**	*Priestia* sp.
**OR948371**	*Acinetobacter* sp.
**OR948361**	*Chitinophaga* sp.
**PP150919**	Enterobacteriaceae
**OR948349**	*Enterobacter* sp.
**OR948339**	*Burkholderia* sp.
**OR948328**	*Pseudomonas* sp.
**OR948401**	*Priestia* sp.
**OR948392**	*Agrobacterium* sp.
**OR948353**	*Pseudomonas* sp.
**OR948054**	*Enterobacter* sp.
**OR948317**	*Enterobacter* sp.
**OR948354**	*Pseudomonas* sp.
**OR948055**	*Enterobacter* sp.
**OR948056**	*Enterobacter* sp.
**OR948358**	*Pseudomonas* sp.

**OR948347**	*Pseudomonas* sp.
**OR948178**	*Enterobacter* sp.
**OR948160**	*Stenotrophomonas* sp.
**OR948179**	*Enterobacter* sp.
**OR948180**	*Enterobacter* sp.
**OR948399**	*Microbacterium* sp.
**OR948390**	*Variovorax* sp.
**OR948380**	*Variovorax* sp.
**OR948359**	*Variovorax* sp.
**OR948337**	*Variovorax* sp.
**OR948326**	*Pseudomonas* sp.
**OR948343**	*Pseudomonas* sp.
**OR948335**	*Cedecea* sp.
**OR948397**	*Pseudomonas* sp.
**OR948388**	*Pseudomonas* sp.
**OR948332**	*Pseudomonas* sp.
**OR948377**	*Pantoea* sp.
**OR948176**	*Stenotrophomonas* sp.
**OR948177**	*Enterobacter* sp.
**OR948367**	*Raoultella* sp.
**OR948356**	*Pantoea* sp.
**OR948175**	*Stenotrophomonas* sp.
**OR948311**	*Pseudomonas* sp.
**OR948378**	*Enterobacter* sp.
**OR948322**	*Pseudomonas* sp.
**PP150914**	Enterobacteriaceae
**OR948357**	*Enterobacter* sp.
**OR948389**	*Enterobacter* sp.
**OR948379**	*Enterobacter* sp.
**OR948368**	*Enterobacter* sp.
**OR948352**	*Massilia* sp.
**OR948075**	*Enterobacter* sp.
**OR948331**	*Achromobacter* sp.
**PP150917**	Enterobacteriaceae
**OR948346**	*Burkholderia* sp.
**PP150899**	*Bacillus* sp.
**OR948111**	*Pseudomonas* sp.
**OR948336**	*Luteibacter* sp.
**PP150918**	Enterobacteriaceae
**OR948398**	*Enterobacter* sp.
**OR948395**	*Bacillus* sp.
**OR948062**	*Enterobacter* sp.
**OR948318**	*Pseudomonas* sp.

**OR948372**	*Burkholderia* sp.
**OR948385**	*Pantoea* sp.
**OR948066**	*Enterobacter* sp.
**OR948067**	*Enterobacter* sp.
**OR948068**	*Enterobacter* sp.
**OR948086**	*Cedecea* sp.
**OR948087**	*Cedecea* sp.
**OR948088**	*Bacillus* sp.
**OR948089**	*Cedecea* sp.
**OR948063**	*Enterobacter* sp.
**OR948064**	*Enterobacter* sp.
**OR948065**	*Enterobacter* sp.
**OR948116**	*Pantoea* sp.
**OR948093**	*Pantoea* sp.
**OR948289**	*Pantoea* sp.
**OR948373**	*Priestia* sp.
**OR948363**	*Burkholderia* sp.
**OR948402**	*Pseudomonas* sp.
**OR948090**	*Cedecea* sp.
**OR948091**	*Bacillus* sp.
**OR948092**	*Bacillus* sp.
**PP150910**	Rhizobiaceae
**OR948350**	*Priestia* sp.
**OR948291**	*Rhodococcus* sp.
**OR948330**	*Agrobacterium* sp.
**PP150911**	*Burkholderia* sp.
**OR948323**	*Pseudomonas* sp.
**OR948351**	*Priestia* sp.
**OR948342**	*Bacillus* sp.
**OR948362**	*Pseudomonas* sp.
**OR948290**	*Pseudomonas* sp.
**PP150915**	Enterobacteriaceae
**OR948341**	*Pseudomonas* sp.
**OR948345**	*Stenotrophomonas* sp.
**OR948334**	*Pseudomonas* sp.
**OR948324**	*Priestia* sp.
**OR948375**	*Priestia* sp.
**PP150922**	Enterobacteriaceae
**OR948387**	*Enterobacter* sp.
**PP150920**	Enterobacteriaceae
**OR948366**	*Stenotrophomonas* sp.
**OR948355**	*Pseudomonas* sp.
**OR948203**	*Pseudomonas* sp.

**PP150907**	Rhizobiaceae
**OR948320**	*Enterobacter* sp.
**OR948292**	*Pseudomonas* sp.
**OR948296**	*Caulobacter* sp.
**OR948094**	*Neobacillus* sp.
**OR948293**	*Pseudomonas* sp.
**OR948294**	*Pseudomonas* sp.
**OR948295**	*Enterobacter* sp.
**OR948298**	*Pseudomonas* sp.
**OR948299**	*Pseudomonas* sp.
**OR948297**	*Massilia* sp.
**OR948286**	*Enterobacter* sp.
**OR948300**	*Paenibacillus* sp.
**OR948303**	*Cedecea* sp.
**OR948301**	*Pseudomonas* sp.
**OR948302**	*Microbacterium* sp.
**OR948306**	*Enterobacter* sp.
**OR948307**	*Priestia* sp.
**OR948304**	*Xanthomonas* sp.
**OR948305**	*Enterobacter* sp.
**OR948308**	*Pseudarthrobacter* sp.
**OR948309**	*Microbacterium* sp.
**OR948310**	*Leifsonia* sp.
**OR948136**	*Flavobacterium* sp.
**OR948150**	*Pseudomonas* sp.
**OR948139**	*Enterobacter* sp.
**OR948156**	*Pantoea* sp.
**OR948143**	*Enterobacter* sp.
**OR948144**	*Enterobacter* sp.
**OR948138**	*Flavobacterium* sp.
**OR948137**	*Flavobacterium* sp.
**OR948141**	*Enterobacter* sp.
**OR948140**	*Enterobacter* sp.
**OR948142**	*Enterobacter* sp.
**OR948365**	*Enterobacter* sp.
